# Benchmarking Boltz-2 for relative kinase-inhibitor interaction patterns across conformational states and resistance mutations

**DOI:** 10.1038/s44386-026-00086-x

**Published:** 2026-10-12

**Authors:** Atsushi Suenaga, Noriaki Okimoto

**Affiliations:** 1 Department of Informatics and Data Science, Sanyo-Onoda City University, Sanyo-Onoda, Yamaguchi, Japan; 2 Laboratory for Computational Molecular Design, RIKEN Center for Biosystems Dynamics Research (BDR), Kobe, Hyogo, Japan; 3 Drug Discovery Molecular Simulation Platform Unit, RIKEN Center for Biosystems Dynamics Research (BDR), Kobe, Hyogo, Japan

**Keywords:** Biochemistry, Biophysics, Computational biology and bioinformatics, Drug discovery, Structural biology

## Abstract

Generative structure-prediction models can directly predict protein-ligand complexes from sequence and ligand information, but their ability to capture pharmacologically relevant kinase-inhibitor interactions remains incompletely understood. We systematically evaluated Boltz-2 using curated biochemical IC_50_ measurements across eight human kinases, nine clinically used inhibitors, and representative resistance mutations. Although predicted log_10_(IC_50_) values showed a compressed dynamic range relative to experimental measurements, mutation-dependent shifts in inhibitor sensitivity, inhibitor selectivity patterns, and overall potency rankings showed substantial agreement with the experimental patterns within the present benchmark. Predicted binding probability showed good discrimination between binder and non-binder pairs within the present benchmark and a consistent inverse relationship with experimental potency. Structural analyses showed that ligand-pose variability was associated with prediction behavior within the evaluated kinase-inhibitor systems, even when kinase-domain structures showed close agreement with the corresponding experimental structures. Larger ligand RMSD values were not associated with larger prediction errors in magnitude, but were linked to systematic shifts in prediction bias, indicating that ligand-pose accuracy and predictive utility are not equivalent measures of Boltz-2 performance. These findings suggest that, within the present benchmark, relative kinase-inhibitor interaction patterns were reproduced more consistently than absolute potency values. We provide a framework for interpreting Boltz-2 outputs within the kinase-inhibitor systems examined here.

## Introduction

Protein kinases regulate diverse cellular processes including proliferation, differentiation, survival, metabolism, and immune signaling. They are therefore considered therapeutically important protein families. Because dysregulated kinase activity plays a central role in cancer and inflammatory disease, numerous small‑molecule kinase inhibitors have been developed over the past two decades^1–4^. Clinically, kinase-targeted therapies have transformed treatment paradigms across multiple malignancies. ABL1 inhibitors marked a major shift in the treatment of chronic myeloid leukemia, and therapies directed at EGFR, HER2, and BRAF now play central roles in lung cancer, breast cancer, and melanoma^5–7^. KIT and PDGFRA are well‑established oncogenic drivers in gastrointestinal stromal tumors and several related neoplasms. RIPK2, in contrast, has gained attention for its role in innate immune and inflammatory signaling^8–10^.

Despite these major advances, prediction of kinase-inhibitor affinity remains challenging. Differences in gatekeeper residues, activation loop mobility, αC‑helix orientation, and solvent‑front architecture can shift ligand preferences, even though kinase catalytic domains share a broadly conserved ATP‑binding fold^11–13^. Single amino‑acid substitutions can also shift drug sensitivity, as seen with resistance mutations such as ABL1 T315I and EGFR T790M/C797S^14–16^.

Traditional computational approaches, including molecular docking, estimate ligand binding poses and relative affinity but are limited by scoring‑function approximations and incomplete treatment of conformational ensembles^17–19^. Recent generative structure-prediction frameworks, including AlphaFold-derived biomolecular modeling approaches, have expanded the scope of computational interaction modeling by jointly reasoning over proteins, nucleic acids, and ligands^20–24^. These advances have enabled high-accuracy reconstruction of biomolecular complex structures. Such frameworks, however, were not primarily designed for direct quantitative prediction of pharmacological affinity or binding probability. Boltz-2 represents a new generation of generative models designed for efficient prediction of biomolecular complexes and interaction propensity^25^. It extends this paradigm by incorporating an affinity-prediction module that directly predicts log_10_(IC_50_) and binding probability from sequence and chemical inputs. A recent study showed that Boltz-2 can classify docking-derived hits with high accuracy, while also raising concerns about the features underlying its affinity predictions^26^. Whether such generative models capture kinase inhibitor recognition, conformational plasticity, and mutation‑dependent rewiring of drug sensitivity remains unclear. Unlike conventional docking or activity‑prediction pipelines, Boltz‑2 may implicitly model distributions of conformational and interaction states.

Kinases serve as a suitable benchmark for evaluating this problem. Although kinase catalytic domains share a conserved ATP‑binding fold, individual kinases differ in gatekeeper composition, activation loop dynamics, αC‑helix positioning, and in the spectrum of clinically observed resistance mutations, all of which influence ligand recognition^11,27–30^.

We assembled a benchmark dataset containing curated biochemical IC_50_ measurements for eight human kinases, nine clinically used inhibitors, and representative oncogenic or resistance variants. This dataset allowed us to evaluate Boltz-2 predictions for kinase-inhibitor recognition, including selectivity, conformational flexibility, and mutation‑dependent drug response. We compared Boltz-2 predictions with experimental inhibitor sensitivity and examined how sequence context and kinase mutations affected prediction performance across different kinase-inhibitor pairs.

## Results

### Assessment of Boltz‑2 inhibitor potency predictions

Boltz‑2 reports predicted log_10_(IC_50_) values, allowing straightforward comparison with experimental measurements. We assessed prediction accuracy by benchmarking these values against curated biochemical potency (IC_50_) for a diverse set of wild‑type kinase-inhibitor pairs. Experimental IC_50_ measurements covered a wide potency range, from sub‑nanomolar (highly active) to micromolar (weakly active), across multiple kinase targets (Supplementary Table S1; see Methods). Three sequence input definitions were tested for kinases: isolated kinase domains, kinase domains with flanking regions, and full-length protein sequences (sequence boundaries are illustrated in Supplementary Fig. S1).

**Fig. 1:**
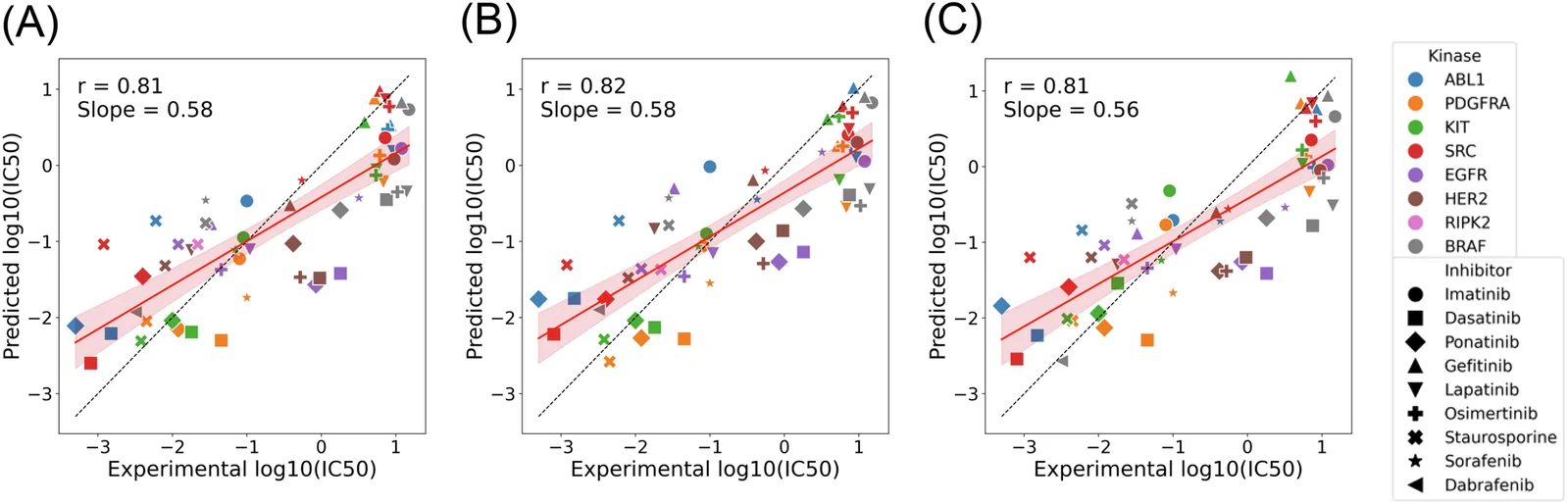
Correlation between experimental and predicted kinase-inhibitor potency values across different sequence input definitions. Scatter plots show predicted versus experimental log_10_(IC_50_) values for 57 kinase-inhibitor pairs using **A** kinase domain only, **B** kinase domain with flanking regions, and **C** full-length protein sequences as model inputs. Colors denote kinase targets and symbol shapes denote inhibitors. Solid red lines indicate linear regression fits with shaded 95% confidence intervals, and dashed lines indicate the identity line (*y* = *x*). Pearson correlation coefficients (*r*) and linear regression slopes are reported for each input definition.

Boltz‑2 predictions correlated strongly with experimental log_10_(IC_50_) values when using isolated kinase domains, kinase domain + flanking regions, or full‑length sequences (Fig. 1). Across three input formats, the overall potency ranking remained stable, with Pearson correlations (*r*) ranging from 0.81 to 0.82. Extending the sequence beyond the isolated kinase domain had little impact on predictive performance. Despite these strong correlations, the predicted values showed systematically reduced variation relative to the experimental measurements. Linear regression of predicted against experimental log_10_(IC_50_) values yielded slopes of 0.580, 0.578, and 0.562 for the kinase domain, kinase domain + flanking-regions, and full-length inputs, respectively. Pair-level bootstrap analyses yielded corresponding 95% confidence intervals of 0.481–0.684, 0.472–0.682, and 0.458–0.670 (Fig. 1; Supplementary Table S2). The confidence intervals were consistently below unity, indicating systematic compression of the experimental potency scale across all three sequence definitions. The bootstrap analyses also supported the stability of the overall associations, with 95% confidence intervals for Pearson correlations of 0.745–0.881, 0.722–0.880, and 0.725–0.871 and corresponding intervals for Spearman correlations of 0.719–0.872, 0.691–0.867, and 0.689–0.861 across the three sequence input definitions. Consistent with the regression analysis, the predicted distributions were narrower than the experimental distribution. The experimental standard deviation was 1.37 log units, whereas the predicted standard deviations were 0.97, 0.98, and 0.96 log units for the kinase domain, kinase domain + flanking-regions, and full-length inputs, respectively. Similarly, the predicted interquartile ranges were 1.47, 1.40, and 1.33 log units, compared with an experimental interquartile range of 2.53 log units.

To determine whether the observed agreement could be explained by simple kinase- or inhibitor-specific potency patterns, we compared Boltz-2 with statistical baselines evaluated using leave-one-pair-out cross-validation (Supplementary Table S3). Among the simple baselines, the inhibitor-mean model showed the strongest performance, with a Pearson correlation of *r* = 0.531, a Spearman correlation of *ρ* = 0.419, an RMSE of 1.168 log units, and an MAE of 0.991 log units. The additive kinase-inhibitor model, which incorporated kinase- and inhibitor-specific effects without an interaction term, achieved *r* = 0.466, *ρ* = 0.473, an RMSE of 1.272 log units, and an MAE of 1.052 log units. In comparison, Boltz-2 achieved Pearson correlations of *r* = 0.806–0.821, Spearman correlations of *ρ* = 0.797–0.817, RMSE values of 0.799–0.834 log units, and MAE values of 0.646–0.686 log units across the three sequence input definitions. Boltz-2 therefore outperformed the simple baselines across all four evaluation metrics, indicating that the observed agreement was not attributable solely to average kinase- or inhibitor-specific potency effects.

Residual analysis further showed that this compression was potency-dependent (Supplementary Fig. S2). Residuals, defined as predicted minus experimental log_10_(IC_50_), were negatively correlated with experimental values across all sequence definitions (Pearson *r* = −0.71 to −0.73). Strong binders with experimental log_10_(IC_50_) values below −1 showed positive mean residuals of 0.48–0.52 log units, indicating underestimation of their potency. In contrast, weak binders and non-binders with experimental log_10_(IC_50_) values of 0 or greater showed negative mean residuals of −0.65 to −0.71 log units, indicating overestimation of their potency. Thus, the compressed prediction range reflected regression toward intermediate potency values rather than a uniform systematic offset.

We examined pair‑specific deviations by visualizing squared prediction errors as kinase-inhibitor heatmaps (Supplementary Fig. S3). Some kinase-inhibitor pairs showed higher errors despite the overall prediction-experiment agreement observed in the benchmark. Pairs involving ABL1, EGFR, and BRAF showed larger deviations than most other kinase-inhibitor pairs. Broad‑spectrum inhibitors such as Dasatinib and Staurosporine exhibited variable error profiles across kinase backgrounds, whereas more selective inhibitors like Gefitinib, Imatinib, and Sorafenib yielded consistently low errors.

Predicted values for ABL1 were similar across the full‑length ABL1 and BCR-ABL1 fusion constructs (Supplementary Table S4). Including dimeric context for EGFR did not improve predictive performance relative to monomeric models. BCR fusion and EGFR dimerization are well‑known drivers of kinase activation. However, their influence on inhibitor potency predictions was minimal in this framework. This pattern is consistent with the limited influence of sequence extension on quantitative potency prediction observed within the present benchmark. The results presented in this section indicate that broad relative potency patterns were reproduced across the kinase-inhibitor systems included in the present benchmark.

### Variability in Boltz‑2 prediction accuracy across kinases and inhibitors

We next examined whether prediction accuracy varied across individual kinases and inhibitors (Supplementary Table S5). ABL1, KIT, PDGFRA, and SRC showed the highest agreement with experimental values, with correlations remaining stable across all sequence definitions (*r* = 0.89–0.95). EGFR and HER2, by contrast, displayed weaker concordance (*r* = 0.47–0.68). Among the inhibitors, Gefitinib, Imatinib, and Dasatinib showed the strongest agreement with experimental values (*r* = 0.84–0.94), whereas broad‑spectrum inhibitors such as Staurosporine and Sorafenib showed weaker agreement (*r* = 0.37–0.65). These patterns show that global potency rankings were broadly reproduced within the present benchmark, although prediction accuracy varied substantially depending on the kinase and inhibitor examined.

We next asked whether differences in prediction accuracy across inhibitors were related to their molecular flexibility. To address this, we compared inhibitor‑wise accuracy with the conformational flexibility index (FI_RMSD) derived from ensemble conformer analysis. Although rigid scaffolds are often assumed to yield more stable predictions, accuracy showed a modest positive trend with flexibility (Supplementary Fig. S4A; *r* = 0.68, *p* = 0.065). Staurosporine, a rigid broad‑spectrum inhibitor, showed relatively weak agreement (*r* = 0.37), whereas more flexible and target‑selective inhibitors such as Lapatinib and Gefitinib displayed stronger correspondence between predicted and experimental values (*r* = 0.74 for Lapatinib, 0.90 for Gefitinib).

The inhibitor‑wise analysis shows that prediction accuracy cannot be explained by conformational flexibility alone, consistent with the importance of conformational ensemble sampling in structure-based prediction^31^. Selective inhibitors generally showed higher agreement with experimental values, whereas broad‑spectrum compounds tended to show weaker correlations. Within this limited inhibitor set, selective inhibitors tended to show higher agreement than broad-spectrum compounds, although this exploratory pattern requires validation in larger and more chemically diverse datasets.

### Assessment of Boltz‑2 binding‑probability predictions

We evaluated binder/non-binder discrimination using Boltz-2’s predicted binding probability within the present benchmark. Kinase-inhibitor pairs with experimental log_10_(IC_50_) < 0 (IC_50_ < 1 μM) were treated as binders, whereas those with log_10_(IC_50_) ≥ 0 were assigned to the non‑binder group. ROC analysis showed strong classification performance across all sequence input definitions (Supplementary Fig. S5; Supplementary Table S2). The AUC values were 0.89 for kinase‑domain inputs, 0.92 for flanking‑region constructs, and 0.92 for full‑length proteins. Pair-level bootstrap analysis yielded corresponding 95% confidence intervals of 0.784–0.962, 0.824–0.981, and 0.825–0.987, respectively. Optimal probability thresholds derived from these ROC curves were 0.29 for kinase‑domain inputs, 0.59 for flanking‑region constructs, and 0.50 for full‑length proteins. The corresponding bootstrap 95% confidence intervals for these threshold estimates were 0.25–0.70, 0.28–0.72, and 0.37–0.70, respectively, indicating greater sampling uncertainty in the specific threshold estimates than in the overall discrimination performance.

**Fig. 2:**
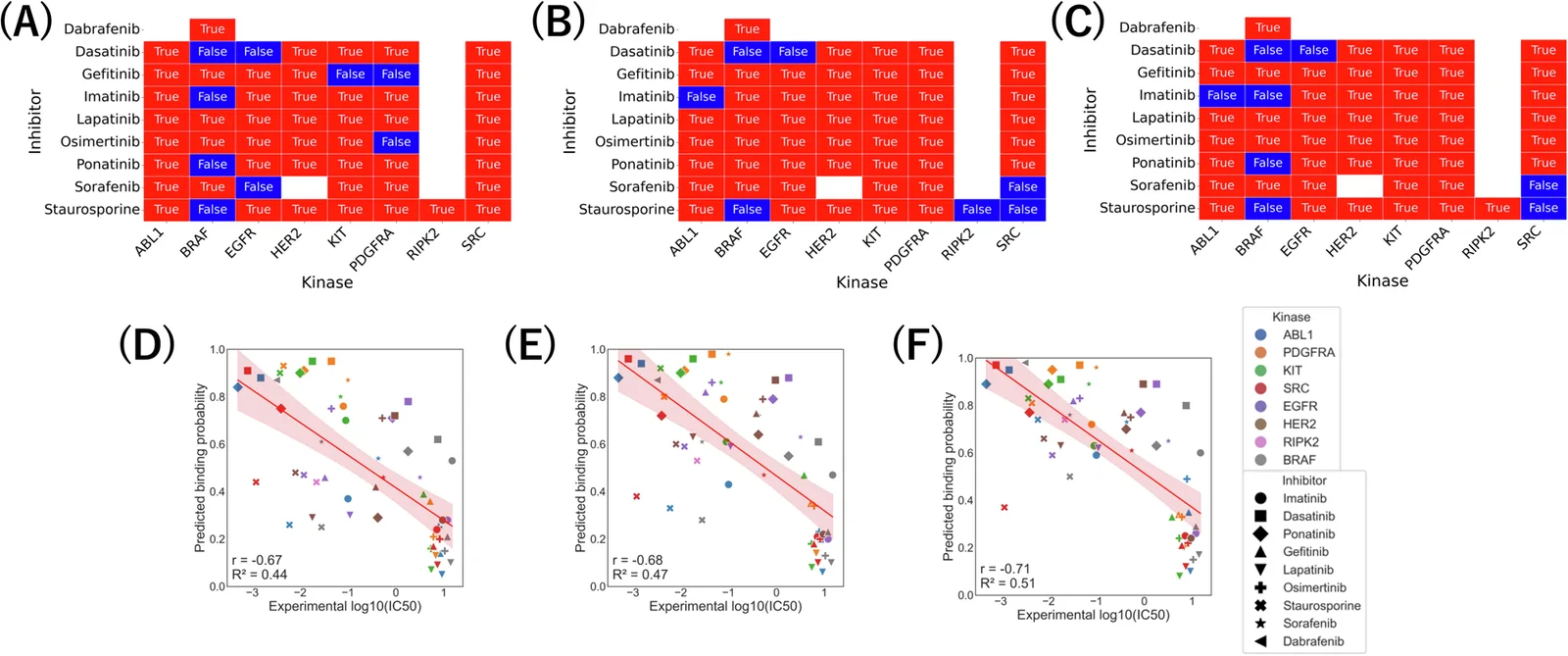
Classification performance and relationship to experimental potency of Boltz-2 binding probability. **A**–**C** Heatmaps summarizing classification outcomes for 57 wild-type kinase-inhibitor benchmark pairs using predicted binding probability thresholds derived from ROC analyses (Supplementary Fig. S5). Predictions were assigned as binder or non-binder using optimal thresholds for **A** kinase domain only, **B** kinase domain with flanking regions, and **C** full-length protein sequences. Red indicates correct classifications and blue indicates incorrect classifications. Experimental binders were defined as log_10_(IC_50_) < 0 (IC_50_ < 1 μM). Scatter plots show the relationship between predicted binding probability and experimental log_10_(IC_50_) values for **D** kinase domain only, **E** kinase domain with flanking regions, and **F** full-length protein sequences. Solid red lines denote linear regression fits with shaded 95% confidence intervals. Pearson correlation coefficients (*r*) and coefficients of determination (*R*²) are reported for each condition.

Using these thresholds, Boltz-2 classified most kinase-inhibitor pairs accurately (Fig. 2A–C), although BRAF showed a higher rate of misclassification. This result indicates that predicted binding probability provided useful binder/non-binder discrimination within the present benchmark. Predicted binding probability also showed an inverse relationship with experimental log_10_(IC_50_), with Pearson correlation coefficients of −0.67 for kinase‑domain inputs, -0.68 for flanking‑region constructs, and −0.71 for full‑length proteins (Fig. 2D–F). This result suggests that predicted binding probability may be useful primarily for binder/non-binder classification while retaining a qualitative association with experimental potency.

Predicted binding probability was also strongly inversely correlated with predicted log_10_(IC_50_) across all sequence definitions, with Pearson correlation coefficients of −0.80, −0.85, and −0.83 for the kinase-domain, kinase domain + flanking regions, and full-length inputs, respectively. This indicates that the two Boltz-2 outputs contained substantially overlapping interaction information within the present dataset. To assess whether combining the two outputs improved binder/non-binder discrimination, we compared leave-one-kinase-out models based on predicted log_10_(IC_50_), binding probability, or both outputs (Supplementary Table S6). Combining the two outputs did not consistently improve classification performance over predicted log_10_(IC_50_) alone, indicating substantial overlap in their binder/non-binder classification information in this dataset.

Stratified analysis revealed clear kinase‑ and ligand‑dependent differences in performance (Supplementary Table S7). Lapatinib, Osimertinib, Gefitinib, and Dasatinib showed strong inverse correlations between predicted probability and experimental log_10_(IC_50_), whereas Sorafenib and Staurosporine showed weaker relationships. Across kinases, KIT, PDGFRA, SRC, and ABL1 displayed high concordance with experimental log_10_(IC_50_), while BRAF, EGFR, and HER2 showed lower correlations. This pattern was consistent with the stratified analysis of predicted potency described above.

A recent benchmarking study by Bret et al. reported that Boltz-2 can classify docking-derived ligand hits across diverse protein targets^26^. Whereas their analysis was based on predefined active and inactive compound sets, here we evaluated binding probability using experimentally measured biochemical IC_50_ in kinase-inhibitor systems. Consistent with the findings of Bret et al.^26^, predicted binding probability showed good discrimination between binder and non-binder kinase-inhibitor pairs within the present benchmark.

### Assessment of mutation‑dependent inhibitor sensitivity

Genetic mutations often alter kinase inhibitor sensitivity^14–16^. To evaluate Boltz-2 predictions for mutation-dependent changes in inhibitor sensitivity, we assembled a benchmark of well-characterized kinase mutants, including ABL1 and EGFR resistance substitutions and the BRAF V600E variant (Supplementary Table S8). We then evaluated Boltz-2 on the mutants, and the full set of prediction outputs is summarized in Supplementary Table S9.

**Fig. 3:**
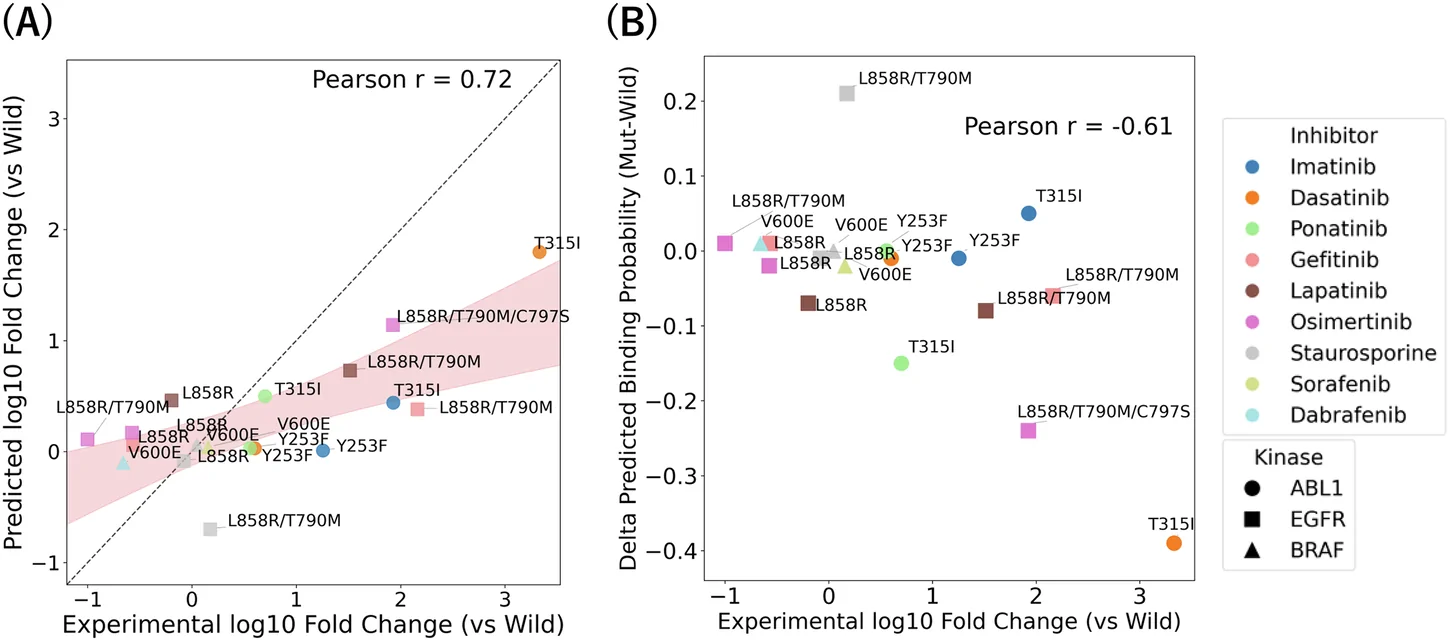
Correlation between experimental and predicted mutation-associated changes in inhibitor sensitivity. **A** Scatter plot comparing experimental and predicted log_10_ fold changes in IC_50_ values for mutant kinases relative to their corresponding wild-type forms. Each point represents a kinase-inhibitor pair. The dashed line indicates the identity line, and red shaded regions denote 95% confidence intervals. Pearson correlation coefficient (*r*) is shown. **B** Relationship between experimental log_10_ fold changes in IC_50_ and corresponding changes in predicted binding probability (mutant minus wild-type). Points are colored by inhibitor and shaped by kinase. Pearson correlation coefficient (*r*) is indicated.

The predicted log_10_(IC_50_) values showed a narrower dynamic range than the experimental measurements, but the mutation-dependent shifts in potency showed substantial agreement with the experimental trends. When we compared experimental and predicted log_10_ fold changes relative to the corresponding wild-type kinases, the agreement was strong (Pearson *r* = 0.72; Fig. 3A). This indicates agreement between predicted and experimental relative changes in inhibitor sensitivity across the kinase mutations examined. For example, the ABL1 gatekeeper mutation T315I produced a clear resistance-associated shift for Dasatinib, with experimental and predicted log_10_ fold changes of +3.33 and +1.80, respectively. Ponatinib was less affected, with smaller experimental and predicted shifts (+0.70 and +0.50).

We next compared the predicted mutation-associated drug-response patterns with clinically established experimental patterns. In EGFR, Gefitinib and Lapatinib lost activity against the T790M mutant, whereas Osimertinib remained effective (experimental log_10_(IC_50_) = −2.35). The predicted log_10_(IC_50_) value for Osimertinib was −1.35, consistent with retained activity against T790M. The subsequent C797S mutation further reduced sensitivity to Osimertinib, increasing the experimental log_10_(IC_50_) from −2.35 to 0.58. The predicted values showed the same direction of change, although the magnitude was smaller (−1.35 to −0.32). For BRAF V600E, the predicted log_10_(IC_50_) value for Dabrafenib was −2.00, consistent with the strong inhibitory activity observed experimentally (log_10_(IC_50_) = −3.16). Dabrafenib was developed to selectively target BRAF V600E, and the predicted value was consistent with this preferential activity.

We next examined whether predicted binding probability reflected mutation-associated changes in inhibitor sensitivity in a manner similar to predicted log_10_(IC_50_) values. Across all kinase-inhibitor pairs, differences in binding probability between mutant and wild-type systems showed a moderate inverse correlation with experimental fold changes in IC_50_ (*r* = −0.61; Fig. 3B). The broader dispersion relative to log_10_(IC_50_) predictions suggests that binding probability is less informative for estimating the magnitude of mutation effects, although the direction of sensitivity changes often agreed with the experimental trends.

### Kinase conformational states in predicted complexes

To assess kinase conformational states in the Boltz-2-predicted complexes, we analyzed the αC-helix and DFG motif conformations in predicted kinase-inhibitor complex structures (Supplementary Table S10). These structural elements are key regulators of kinase activity and define canonical active (αC-IN/DFG-IN) and inactive (DFG-OUT) conformations.

Across the kinase-inhibitor systems examined, the predicted structures exhibited both active-like and inactive-like conformational states. Active-like conformations (αC-IN/DFG-IN) were consistently observed for several kinase-type I inhibitor pairs, including Dasatinib-ABL1, Gefitinib-EGFR, and Osimertinib-EGFR, in agreement with experimentally determined structures. Inactive-like conformations characterized by DFG-OUT states were also observed for type II inhibitor complexes such as Imatinib-ABL1, Imatinib-PDGFRA, Imatinib-KIT, Ponatinib-KIT, Ponatinib-SRC, and Sorafenib-BRAF, consistent with experimentally determined structures.

The predicted conformational landscape did not strictly follow inhibitor class. While type II inhibitors were generally associated with DFG-OUT conformations, several type I inhibitors were observed in both DFG-IN and DFG-OUT states depending on the kinase context. For example, Staurosporine, Gefitinib, Osimertinib, Lapatinib, and Dasatinib exhibited kinase-dependent variability in predicted conformations, adopting active-like or inactive-like states across different targets. This kinase-dependent variability is consistent with previous structural analyses showing that some type I inhibitors can bind to both DFG-IN and DFG-OUT conformations^32^.

Agreement between predicted and experimental conformational states was observed for many kinase–inhibitor pairs with available experimental structures. For kinase-inhibitor pairs lacking corresponding experimental structures, the predicted conformational states included both active-like (αC-IN/DFG-IN) and inactive-like (DFG-OUT) conformations, depending on kinase and inhibitor context. These findings indicate that the predicted complexes showed conformational states consistent with context-dependent inhibitor recognition across the systems examined.

### Structural accuracy and ligand-pose variability in predicted complexes

**Fig. 4:**
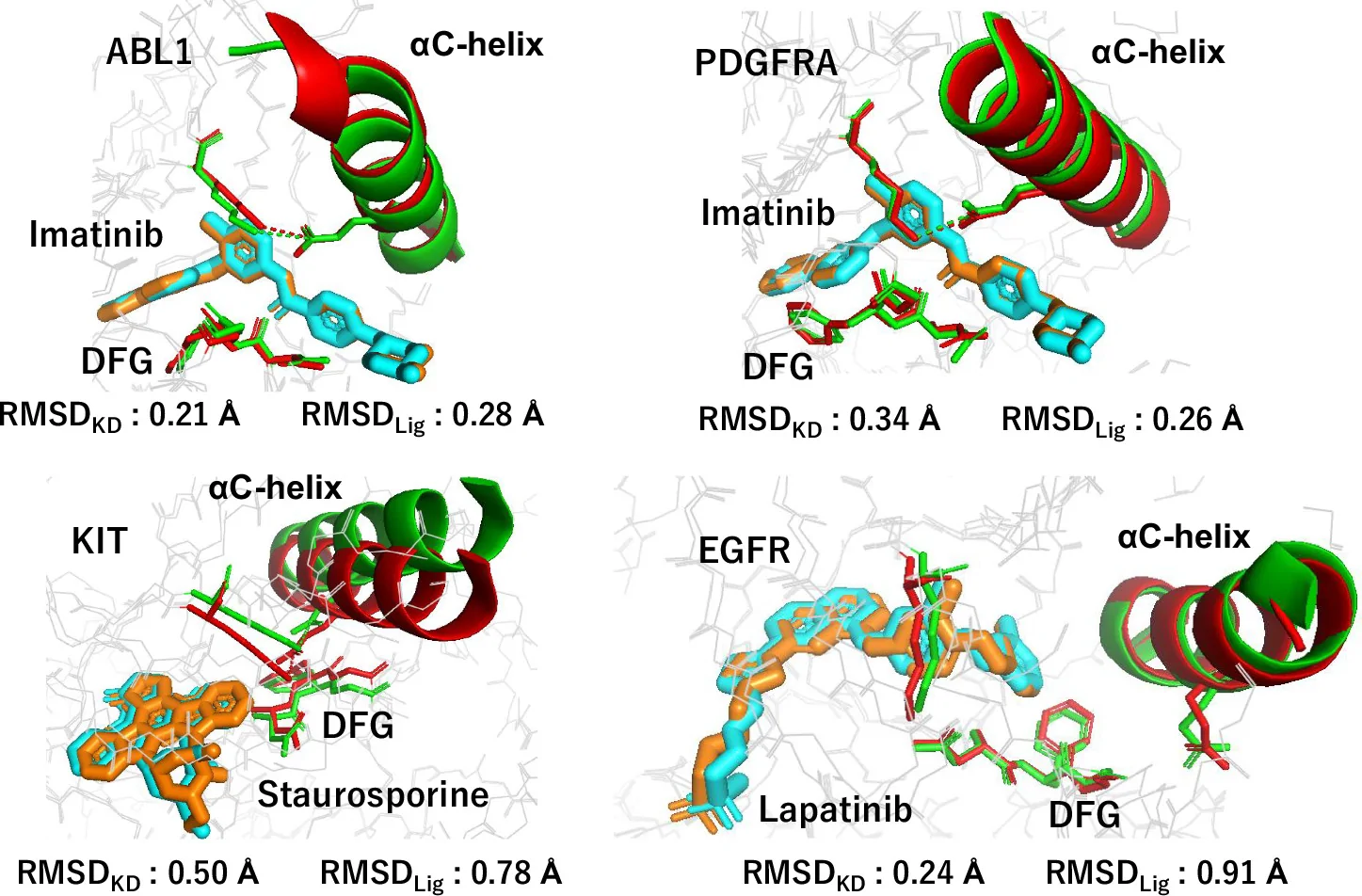
Comparison of experimental and predicted kinase-inhibitor complex structures. Kinase domains are shown as cartoons and inhibitors as sticks. Experimental structures are colored green for kinases and orange for ligands, whereas predicted structures are colored red for kinases and cyan for ligands. The αC-helix and DFG motif are highlighted for reference. Root-mean-square deviations for the kinase domain (RMSD_KD_) and ligand (RMSD_Lig_) are reported for each complex.

To characterize structural agreement between the predicted kinase-inhibitor complexes and corresponding experimental structures, we compared the models using root-mean-square deviation (RMSD) metrics for both kinase domains (RMSD_KD_) and ligand binding poses (RMSD_Lig_) (Supplementary Table S11). Structural analyses were performed using representative structures generated from kinase domain-only sequence inputs (see Methods). Representative structures showed close agreement with the global kinase folds and key local features of the corresponding experimental structures (Fig. 4). Quantitative analysis further showed consistently low kinase-domain RMSD values (RMSD_KD_ generally below 0.5 Å), whereas ligand binding poses exhibited substantially broader variability across kinase-inhibitor pairs (RMSD_Lig_ = 0.25–3.29 Å) (Supplementary Table S11; Supplementary Figs. S6, S7).

**Fig. 5:**
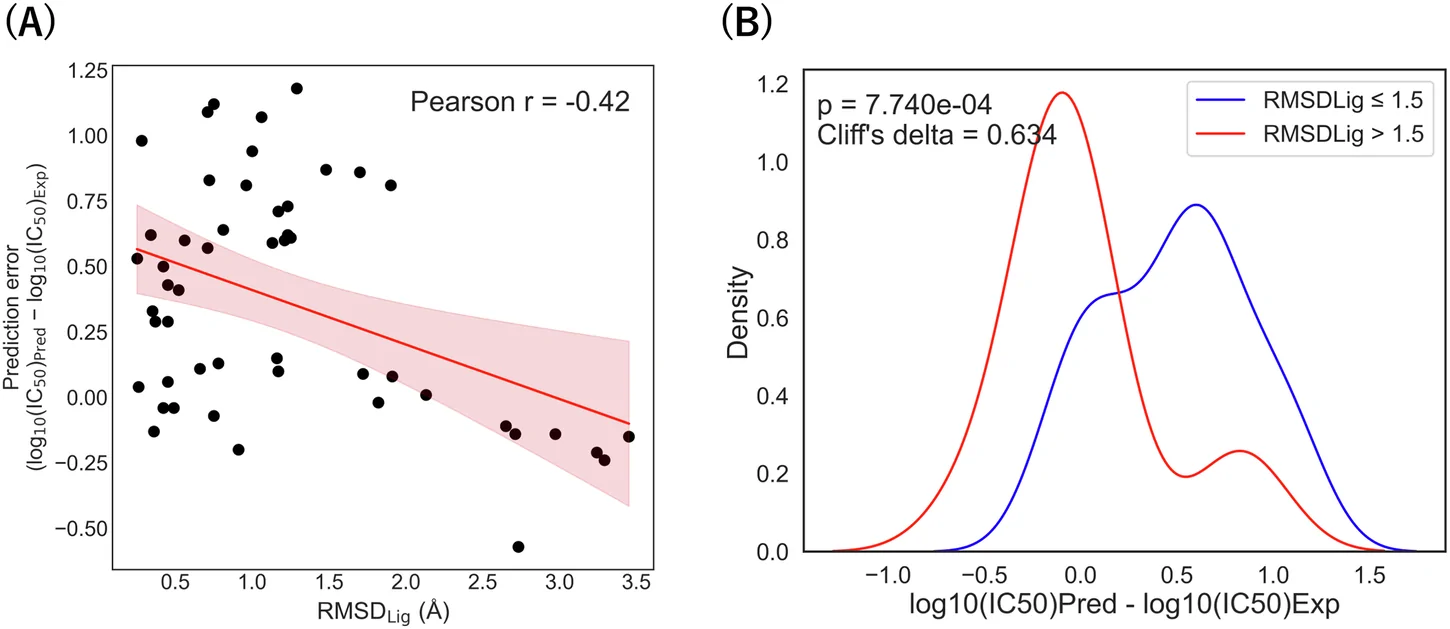
Relationship between ligand RMSD and quantitative prediction error. **A** Scatter plot showing ligand RMSD (RMSD_Lig_) versus prediction error [log_10_(IC_50_)_pred_ − log_10_(IC_50_)_exp_] across kinase-inhibitor pairs. The red line denotes the linear regression fit with shaded 95% confidence intervals. Pearson correlation coefficient (*r*) is indicated. **B** Distribution of prediction errors stratified by ligand RMSD using a threshold of 1.5 Å. Statistical significance was assessed using the Mann–Whitney U test (*p* value shown), and effect size was quantified using Cliff’s delta.

We next asked whether more accurate ligand binding poses were associated with improved potency prediction. To assess this, we analyzed the relationship between RMSD_Lig_ and prediction error, defined as predicted minus experimental log_10_(IC_50_) values. We detected a moderate negative correlation (Pearson *r* = −0.42; Fig. 5A), indicating an exploratory association between larger ligand-pose deviations and a shift toward potency overestimation within this structural subset. When predicted complexes were grouped using an RMSD_Lig_ threshold of 1.5 Å, the two groups showed significantly different prediction‑error distributions (Fig. 5B; Mann–Whitney U test, *p* = 7.74 × 10⁻⁴; Cliff’s *δ* = 0.634). Complexes with lower RMSD_Lig_ values (≤1.5 Å) showed broader prediction-error distributions with a tendency toward mildly positive errors, whereas higher RMSD_Lig_ structures (>1.5 Å) exhibited distributions shifted toward more negative values. Within the evaluated structural subset, larger RMSD_Lig_ values were not associated with larger prediction errors in magnitude but were accompanied by a shift in error sign toward potency overestimation.

To examine variability across prediction runs, we analyzed multiple predicted poses for each kinase-inhibitor pair. We performed 10 Boltz‑2 runs per pair, and each system produced a set of 10 poses with markedly different RMSD_Lig_ values. For example, Lapatinib showed clear variability in predicted binding poses across runs (Supplementary Fig. S8). Even when RMSD_Lig_ values were large, the structural differences were often localized, and the inhibitor occupied a similar region of the active site as the experimental pose. This indicates that substantial ligand-pose deviations can arise from local differences in ligand placement without altering the overall binding mode.

Finally, to provide direct structural baselines, we compared ligand-pose accuracy among Boltz-2, AlphaFold 3, and Chai-1 across 12 wild-type kinase-inhibitor complexes with matched experimental co-crystal structures (Supplementary Table S12). The median RMSD_Lig_ values were 0.80 Å for Boltz-2, 0.95 Å for AlphaFold 3, and 1.87 Å for Chai-1, with corresponding mean values of 0.92, 0.93, and 3.53 Å, respectively. Eleven of the 12 representative structures generated by Boltz-2 and AlphaFold 3 had RMSD_Lig_ values below 1.5 Å, compared with five of the 12 Chai-1 structures. The paired RMSD_Lig_ distributions of Boltz-2 and AlphaFold 3 did not differ significantly (Wilcoxon signed-rank test, *p* = 0.791). Boltz-2 and AlphaFold 3 showed broadly similar RMSD_Lig_ distributions, whereas Chai-1 exhibited greater complex-dependent variability, including substantially larger deviations for the three Imatinib complexes. These results indicate that ligand-pose RMSD_Lig_ values varied across the three models and individual kinase-inhibitor systems in this limited kinase-focused comparison.

We next assessed potential structural training exposure using the stated Boltz-2 structural training cutoff of 1 June 2023. Exact wild-type human kinase-inhibitor complexes released before this cutoff were identified for 11 of the 12 kinase-inhibitor pairs, with the number of pre-cutoff exact complexes ranging from one to six among these pairs (Supplementary Table S13). KIT-Staurosporine was the only pair for which no pre-cutoff exact wild-type complex was identified. Thus, 11 of the 12 structural benchmark pairs had potential direct structural training exposure. Accordingly, the ligand-pose comparisons for these systems should not be interpreted as evidence of de novo pose prediction, but rather as assessments of reconstruction accuracy and consistency within largely structurally familiar kinase-inhibitor space.

We additionally examined the record-level provenance of the seven kinase-inhibitor pairs supported by at least 10 curated IC_50_ measurements (Supplementary Table S14). The underlying measurements were derived from PubChem and/or BindingDB records, with corresponding BindingDB DOI and ChEMBL assay/source identifiers available for most records. Many of these measurements had been publicly recorded well before the release of Boltz-2, including numerous records dating from 2009 to 2022. Because PubChem, ChEMBL, and BindingDB were also used as sources for Boltz-2 affinity training, prior exposure to some of these measurements or kinase-inhibitor relationships cannot be excluded. However, actual inclusion of individual records in the Boltz-2 affinity-training dataset could not be determined from the publicly reported training information.

### Comparison with established affinity-scoring baselines

To assess whether the relative potency trends observed in the Boltz-2 predictions were also recovered by established structure-based affinity-scoring approaches, we compared Boltz-2 with RTMScore, MMGBSA, and MMPBSA using the same 57 wild-type kinase-inhibitor pairs (Supplementary Table S15). Across all 57 pairs, Boltz-2 showed the strongest agreement with experimental log_10_(IC_50_) values, with Pearson and Spearman correlations of *r* = 0.820 and *ρ* = 0.816, respectively. RTMScore showed the next strongest global association (*r* = −0.611, *ρ* = −0.624), where the negative sign reflects the opposite score direction because higher RTMScore values indicate stronger predicted binding. MMPBSA showed weaker correlations (*r* = 0.385, *ρ* = 0.304), followed by MMGBSA (*r* = 0.268, *ρ* = 0.250) (Supplementary Table S16).

Kinase-wise analysis showed a similar overall pattern. Boltz-2 exhibited a higher Pearson correlation than each of the three affinity-scoring baselines for all seven kinases with sufficient numbers of inhibitor pairs, although baseline performance varied substantially among individual kinases (Supplementary Table S17). These comparisons indicate that, within the present benchmark, the Boltz-2 affinity predictions retained relative potency information that was not recovered to the same extent by the three established rescoring approaches applied to the same predicted complexes.

### Sequence‑context effects on predicted kinase-inhibitor interactions

Analysis of the full prediction dataset (Supplementary Table S4) showed that EGFR and HER2 displayed marked differences in binding probability estimates depending on sequence context. For example, the binding probability for EGFR-Gefitinib increased from 0.46 (KD) to 0.82 ((+)-KD-(+)), and HER2-Gefitinib showed a similar shift from 0.42 (KD) to 0.73 ((+)-KD-(+)). In contrast, predicted log_10_(IC_50_) values showed much smaller changes across sequence definitions. To further investigate the effect of sequence context on binding probability predictions, we analyzed EGFR using four input definitions. In addition to the sequence definitions evaluated above, EGFR was further examined using kinase domains with either an N-terminal [(+)-KD] or a C-terminal [KD-(+)] extension alone.

**Fig. 6:**
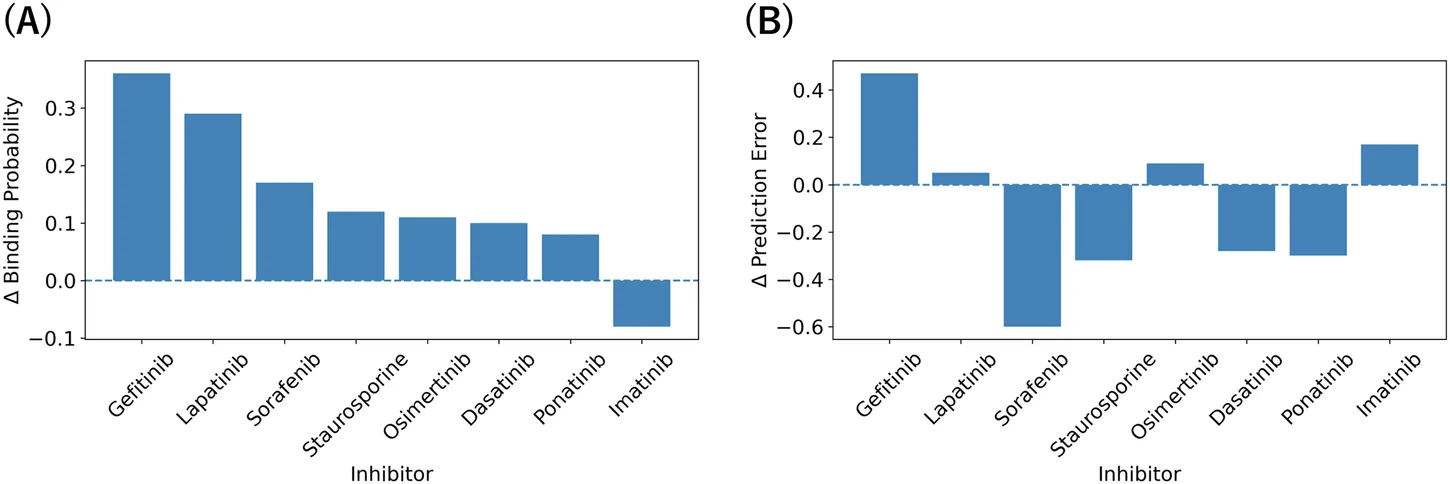
Effects of sequence context on prediction outputs for EGFR inhibitor systems. **A** Changes in predicted binding probability (ΔBP) upon extending the EGFR input sequence from the kinase-domain-only (KD) definition to the sequence-extended (+)-KD-(+) definition. ΔBP values were calculated as the difference between BP_(+)-KD-(+)_ and BP_KD_. **B** Changes in prediction error under the same sequence-context extension. Prediction-error changes were calculated as [log_10_(IC_50_)_pred_ − log_10_(IC_50_)_exp_]_(+)–KD–(+)_ − [log_10_(IC_50_)_pred_ − log_10_(IC_50_)_exp_]_KD_, where negative values indicate improved agreement with experimental measurements after sequence extension.

Figure 6 summarizes the inhibitor‑specific effects of sequence‑context extension by plotting Δ(binding probability) (ΔBP) and Δ(prediction error) values for EGFR, calculated as the difference between the (+)-KD-(+) and KD inputs. Most inhibitors showed positive ΔBP values, with Gefitinib and Lapatinib displaying the largest increases and Imatinib being the only clear exception. In contrast, changes in Δ(prediction error) varied across inhibitors without a consistent direction, suggesting that sequence-context extension had a stronger effect on binding probability estimates than on quantitative affinity prediction.

**Fig. 7:**
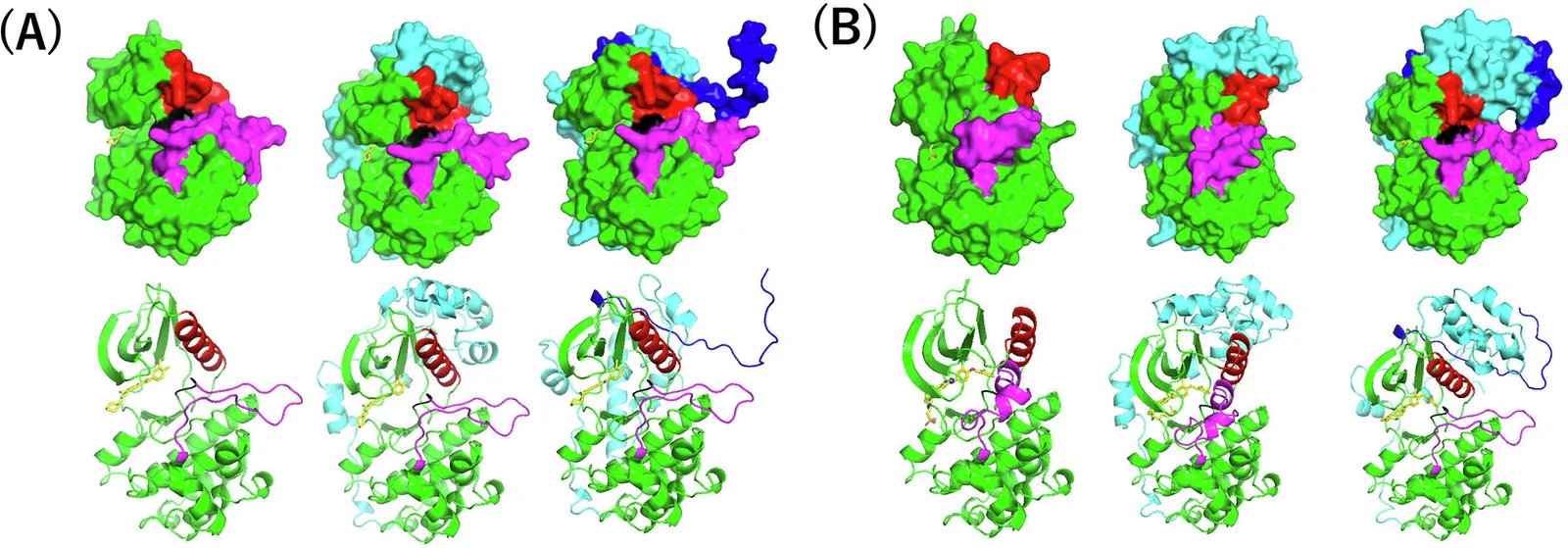
Structural comparison of EGFR inhibitor complexes under different sequence-context definitions. **A** EGFR-Gefitinib and **B** EGFR-Lapatinib predicted structures generated using three sequence input definitions: kinase domain only (KD, left), kinase domain with C-terminal extension [KD-(+), center], and kinase domain with both N- and C-terminal extensions [(+)-KD-(+), right]. Upper panels show surface representations and lower panels show cartoon representations of the predicted structures. The kinase domain is colored green, the αC-helix red, the DFG motif black, the activation loop magenta, and inhibitors yellow. Additional N-terminal regions included in (+)-KD-(+) are shown in blue, whereas additional C-terminal regions included in KD-(+) and (+)-KD-(+) are shown in cyan.

We next examined the structural origin of these changes by comparing predicted EGFR-Gefitinib and EGFR-Lapatinib complexes across the sequence‑context definitions (Fig. 7). Adding flanking regions introduced folded structural elements adjacent to the kinase domain, most prominently around the αC‑helix and activation loop. Extension of the C‑terminal region [KD-(+)] produced the largest binding probability increases for both inhibitors (Supplementary Table S18). These binding probability shifts did not coincide with changes in αC or DFG conformational states, which remained stable across sequence definitions. The sequence extensions instead altered the local environment around the active site.

HER2 showed sequence‑context‑dependent increases in predicted binding probability similar to those observed in EGFR (Supplementary Fig. S9). In both systems, the extended C‑terminal regions formed additional folded structural elements surrounding the kinase‑domain C‑lobe and activation loop. These structural features were observed without substantial changes in the overall kinase domain conformation.

ABL1-Dasatinib displayed only limited sequence‑context effects. The extended regions formed folded structural elements outside the kinase domain and did not closely associate with the activation loop or αC‑adjacent regions. BRAF-Sorafenib also showed minimal changes following sequence extension, and the additional sequence segments remained spatially separated from the active‑site environment (Supplementary Fig. S9). These comparisons suggest that sequence-context sensitivity may depend on how flanking regions are positioned relative to the kinase domain.

EGFR dimer models were generated using sequence‑extended inputs and compared with experimental EGFR dimer structures (Supplementary Fig. S10). In the predicted dimers, the extended C‑terminal regions formed organized structural elements surrounding the kinase‑dimer interface and activation‑loop‑associated regions. The predicted dimers did not reproduce the trans‑autophosphorylation geometries established experimentally, but the overall organization resembled asymmetric ErbB‑family dimer architectures, such as those observed in EGFR and HER2, that are associated with receptor activation and regulatory‑tail positioning^33–35^.

## Discussion

In this study, we systematically evaluated Boltz-2 across a focused panel of kinase-inhibitor systems. Although predicted log_10_(IC_50_) values showed a compressed dynamic range compared with experimental measurements, mutation-dependent shifts, inhibitor selectivity patterns, and overall potency rankings showed substantial agreement with the experimental patterns within the present benchmark. The additional regression, distributional, and residual analyses further showed that this behavior reflected systematic compression toward intermediate potency values rather than a uniform offset. Strong binders were generally predicted as weaker, whereas weak binders and non-binders were predicted as stronger. These observations indicate that, within the present benchmark, relative kinase-inhibitor interaction patterns were reproduced more consistently than absolute potency values.

Importantly, the observed agreement was not explained solely by simple kinase- or inhibitor-specific potency patterns. In leave-one-pair-out comparisons, the strongest simple baseline, based on inhibitor-mean potency, achieved a Pearson correlation of *r* = 0.531 and an RMSE of 1.168 log units, whereas Boltz-2 achieved correlations of *r* = 0.806–0.821 and RMSE values of 0.799–0.834 log units across sequence definitions. The additive kinase-inhibitor model also performed less well than Boltz-2, indicating that the predictions retained information beyond independent kinase- and inhibitor-level average effects.

The comparison with established affinity-scoring methods further supported this interpretation. Using the same Boltz-2-predicted kinase-domain complexes as structural inputs, Boltz-2 showed stronger global correlations with experimental log_10_(IC_50_) values than RTMScore, MMGBSA, and MMPBSA. In the kinase-wise analysis, Boltz-2 also showed the highest Pearson correlation among the four methods for all seven kinases evaluated. Because the three baseline methods were applied as rescoring approaches to the same Boltz-2-predicted complexes, these comparisons do not constitute fully independent end-to-end benchmarks. Nevertheless, within the present dataset, the results indicate that the Boltz-2 affinity predictions retained relative potency information that was not recovered to the same extent by these established structure-based rescoring approaches.

The reproduction of mutation-dependent changes in inhibitor sensitivity provides an additional example of the relative interaction patterns retained within the present benchmark. Mutation-associated shifts in inhibitor response showed substantial agreement with the experimental trends, including clinically established resistance mechanisms involving EGFR T790M/C797S and ABL1 T315I. Within the present benchmark, these mutation-dependent shifts suggest potential utility for comparative analyses in which relative changes in drug response are of primary interest.

A second notable finding was the utility of the binding probability output for binder/non-binder classification and qualitative assessment of interaction strength. Consistent with previous reports^26^, binding probability showed good discrimination between binder and non-binder kinase-inhibitor pairs within the present benchmark. Although binding classification and continuous affinity regression are implemented as distinct prediction tasks in Boltz-2 training^25^, binding probability was strongly associated with predicted log_10_(IC_50_) in the present dataset. Moreover, combining the two outputs did not consistently improve binder/non-binder classification over predicted log_10_(IC_50_) alone. These findings indicate that the two outputs contained substantially overlapping, although not identical, interaction information within the present dataset. In addition, binding probability showed a consistent inverse relationship with experimental potency and qualitative agreement with mutation-associated sensitivity trends across the backgrounds examined. Binding probability may therefore be most appropriately interpreted as a normalized classification-oriented output that also retains a qualitative association with potency, rather than as an independent quantitative affinity measure. From a practical perspective, the normalized output range of binding probability facilitates threshold-based classification, although the optimal threshold varied across sequence input definitions. In contrast, the compressed dynamic range of predicted log_10_(IC_50_) values makes the selection of classification thresholds less straightforward.

The systematic compression of predicted log_10_(IC_50_) values may arise from several non-exclusive factors. First, the affinity-prediction component may be calibrated to minimize error across heterogeneous training examples, favoring predictions closer to the center of the training distribution when the available structural information is insufficient to support extreme potency estimates. Second, the affinity-related labels used during Boltz-2 training include multiple endpoint types, such as IC_50_, *K*_*i*_*, K*_*d*_, AC_50_, EC_50_, and XC_50_, which do not represent equivalent biochemical quantities and may differ in scale and experimental context. Training against these heterogeneous measurements may contribute to the observed preservation of broad rank relationships while limiting absolute calibration to any single endpoint. Third, static protein-ligand structures do not explicitly encode several determinants of measured IC_50_, including ATP and substrate concentrations, kinetic mechanisms, protein constructs, incubation conditions, and assay formats. These factors may help explain why relative potency patterns were retained more consistently than the full magnitude of absolute potency differences. However, the present benchmark cannot determine the individual contribution of the model architecture, affinity head, training objective, label heterogeneity, or assay conditions to the observed compression.

Structural analyses provided additional insight into the factors underlying prediction performance. Whereas kinase-domain structures showed consistently low RMSD values relative to the experimental structures, ligand binding poses showed substantially greater variability across kinase-inhibitor pairs. In the evaluated structural subset, RMSD_Lig_ was associated with shifts in prediction-error distributions, suggesting a possible relationship between local differences in ligand placement and potency predictions even when the overall kinase fold showed close agreement with the corresponding experimental structure. Further analyses of the ten independent Boltz-2 predictions showed that large RMSD_Lig_ values often arose from localized structural differences, while retaining a broadly similar binding mode to the experimental pose within the active site. In this analysis, larger RMSD_Lig_ values were not associated with larger prediction errors in magnitude, but were accompanied by a systematic shift in the sign of the error toward potency overestimation.

The direct comparisons with AlphaFold 3 and Chai-1 provided external structural baselines for the ligand-pose analysis. Across the 12 wild-type kinase-inhibitor complexes examined, Boltz-2 and AlphaFold 3 showed broadly similar RMSD_Lig_ distributions, whereas Chai-1 exhibited greater complex-dependent variability and substantially larger deviations for the three Imatinib complexes. At the same time, Chai-1 reproduced several other ligand poses with sub-angstrom accuracy, indicating that its performance was strongly dependent on the individual kinase-inhibitor system rather than uniformly reduced across the benchmark. These findings further show that ligand-pose RMSD distributions can differ substantially across models and inhibitor contexts. However, the comparison was limited to a small set of wild-type kinase complexes with available matched co-crystal structures, and the sampling depth and representative-structure selection procedures differed among the three models. The results therefore provide a restricted model comparison and should not be interpreted as establishing general equivalence or superiority across broader protein-ligand prediction tasks.

An unexpected observation was that inhibitors with higher FI_RMSD values tended to show better agreement between predicted and experimental potency values. This trend contrasts with the intuitive expectation that conformationally rigid inhibitors should be easier to predict than highly flexible molecules. One possible explanation is that flexible inhibitors may generate a broader range of alternative binding poses during prediction. A larger ensemble of plausible poses may increase the likelihood that some are more informative for potency prediction, even when their RMSD_Lig_ values differ substantially from those of the experimental structure. In contrast, conformationally rigid inhibitors may allow fewer alternative binding solutions, making prediction outcomes more dependent on the accuracy of a single dominant pose. Although this trend requires validation using larger datasets, our observation contrasts with the conventional view that increasing conformational flexibility generally increases the difficulty of structure-based prediction^17–19^. Within the present inhibitor set, these observations suggest that RMSD_Lig_ alone may not fully reflect the relationship between binding-pose variation and potency prediction, particularly for flexible inhibitors.

More broadly, structural accuracy and predictive utility should not necessarily be regarded as equivalent measures of Boltz-2 performance. In several analyses, deviations from experimental ligand poses were not consistently associated with poorer prediction outcomes, and larger RMSD_Lig_ values were not always accompanied by reduced predictive performance. Similar observations have been reported in deep learning-based docking and cofolding frameworks, where useful predictive information can be retained despite inaccuracies in detailed ligand placement^36–38^. In particular, a recent Boltz-2 benchmark of docking hits reported that binding classification performance could remain strong even when predicted ligand poses deviated from the expected docking poses, while also raising concerns about the hidden features underlying Boltz-2 affinity predictions^26^. Our findings provide exploratory evidence for a relationship between ligand-pose variability and prediction behavior within the kinase-inhibitor systems examined, based on both RMSD_Lig_ and FI_RMSD analyses.

The selective sequence-context effects observed for EGFR and HER2 were particularly notable. Sequence extension produced substantial increases in predicted binding probability in both kinases. Structural analyses showed that the added sequences frequently formed folded elements adjacent to the activation loop and αC-helix, thereby altering the local structural environment surrounding the active site. In several cases, these elements interacted with the N-lobe and activation loop rather than directly contacting the ATP-binding pocket, suggesting an indirect effect on the predicted binding state. Interestingly, sequence-context effects were substantially more pronounced for binding probability than for predicted log_10_(IC_50_) values. Because binding probability and predicted log_10_(IC_50_) are supervised through related but distinct prediction tasks during Boltz-2 model training^25^, the stronger sequence-context dependence observed for binding probability suggests that the two outputs may rely on different structural features. One possibility is that binding probability is influenced by structural features extending beyond the ligand-binding pocket, whereas predicted log_10_(IC_50_) values may be driven more strongly by the local binding-site environment. In contrast, sequence extension produced only limited changes in binding probability in the other kinases examined in this study. For example, structural analyses of ABL1 and BRAF showed that the folded elements formed by the added sequences remained spatially separated from the activation-loop and αC-helix regions. These observations are broadly consistent with the distinct regulatory architectures of these kinase families. In ErbB-family kinases, regions flanking the kinase domain contribute directly to receptor activation and signaling through mechanisms involving C-terminal regulatory elements, receptor dimerization, and trans-autophosphorylation^33–35^. By contrast, regulation of ABL1 and BRAF is mediated primarily through SH2/SH3-dependent autoinhibition^39^ or 14-3-3-dependent interactions^40^, which may be less directly coupled to the local active-site environment. These observations raise the possibility that higher-order structural organization may influence Boltz-2 binding-probability estimates in selected kinase systems, although this interpretation requires validation in a substantially larger and more diverse dataset.

Collectively, these findings suggest several practical considerations for the use of Boltz-2 in kinase-inhibitor studies. Across multiple analyses within the present benchmark, relative trends were generally more consistent than absolute potency estimates. In addition, within the kinase-inhibitor systems examined, comparative analyses including inhibitor ranking, mutation-associated changes in drug response, and identification of likely binders showed potential practical utility. Multiple independent Boltz-2 predictions sometimes produced different ligand poses, suggesting that a single predicted structure may not fully represent the range of plausible kinase-inhibitor configurations generated by Boltz-2. This consideration may be particularly important for inhibitors that exhibit substantial pose variability. The results therefore suggest that evaluating multiple independent Boltz-2 predictions for the same kinase-inhibitor pair may provide additional insight beyond that obtained from a single predicted structure.

Several limitations should be considered when interpreting the present findings. The analyses were restricted to kinase-inhibitor systems, and it remains unclear whether the relationships observed between structural accuracy, ligand-pose variability, and prediction behavior are generalizable to other target classes. Although large-scale evaluations of Boltz-2 have been reported previously^25,26^, the present study was intentionally designed as a focused analysis of kinase-inhibitor systems to enable detailed evaluation of sequence context, resistance mutations, conformational states, and ligand-pose variability. The benchmark size also limited the number of observations available for kinase-wise, inhibitor-wise, mutation-specific, and structure-prediction relationship analyses. These subgroup analyses should therefore be regarded as exploratory observations within the selected kinase-inhibitor panel, and their stability will require evaluation in larger and more diverse datasets. The findings should therefore be regarded as complementary to broader benchmarks rather than as a comprehensive assessment of general protein-ligand prediction performance. Protein kinases provide a particularly suitable benchmark for this type of analysis because of the extensive availability of experimental potency data, structural information, and clinically relevant resistance mutations. The mutation analysis was also restricted to selected, well-characterized variants in ABL1, EGFR, and BRAF. Because these canonical resistance and oncogenic mutations have been extensively studied in public structural and pharmacological resources, the present results do not establish generalization to rare, less-characterized, or previously unseen mutations. The present benchmark also did not systematically evaluate less-studied kinases, noncanonical or allosteric inhibitors, matched active-inactive analog series, or prospective and temporally separated test settings, and the current results should not be interpreted as establishing performance in these more challenging contexts. Future studies extending similar analyses to additional protein families, more diverse mutation and inhibitor classes, and independently curated prospective or temporally separated benchmarks will be important for determining the broader applicability of these observations.

A further limitation is that potential overlap between the present benchmark and the data used to train Boltz-2 could not be fully evaluated. Several of the kinase-inhibitor pairs and resistance mutations examined here are well characterized and are represented extensively in public structural and pharmacological resources. Using the stated Boltz-2 structural training cutoff of 1 June 2023, we identified at least one exact wild-type kinase-inhibitor complex released before the cutoff for 11 of the 12 structural benchmark pairs, indicating potential direct structural training exposure for most of these systems. KIT-Staurosporine was the only pair for which no pre-cutoff exact wild-type complex was identified. Public availability before the cutoff does not establish that an individual structure was actually included in Boltz-2 training, but direct structural exposure cannot be excluded for these 11 pairs. Accordingly, the structural comparisons should be interpreted as assessments of reconstruction accuracy and consistency within largely structurally familiar kinase-inhibitor space rather than as evidence of de novo ligand-pose prediction. Consequently, the observed performance should not be interpreted as evidence of fully de novo or out-of-distribution generalization. Future evaluations using temporally separated, prospective, or explicitly low-redundancy benchmarks will be required to distinguish recovery of previously represented relationships from generalization to less characterized systems.

Potential overlap also applies to the experimental affinity data used in the present benchmark. The Boltz-2 affinity module was trained using continuous affinity measurements curated from PubChem, ChEMBL, and BindingDB, which overlap directly with the public databases used here. Our record-level audit of the seven kinase-inhibitor pairs supported by at least 10 curated IC_50_ measurements showed that many of the measurements contributing to the representative median values had been publicly recorded well before the release of Boltz-2 (Supplementary Table S14). This provenance audit was restricted to the seven most data-rich pairs and therefore does not constitute a complete measurement-level audit of all 57 benchmark pairs. Therefore, prior exposure to individual measurements or to the corresponding kinase-inhibitor relationships cannot be excluded. However, the Boltz-2 publication does not provide a measurement-level training-data manifest or an explicit temporal cutoff for the affinity-training dataset, and public availability alone does not establish that a given measurement was actually used for training. Accordingly, the observed agreement between predicted and experimental potency for these well-characterized systems should not be interpreted as evidence of de novo or out-of-distribution affinity prediction. Nor can potential training overlap be assumed to account causally for the observed prediction accuracy without controlled retraining or ablation experiments.

Another consideration is that Boltz-2 performance was evaluated using experimentally determined IC_50_ values. This choice was motivated by the fact that Boltz-2 reports predicted log_10_(IC_50_) values, enabling direct comparison between model predictions and experimental measurements. However, because IC_50_ is assay-dependent and the Boltz-2 affinity model was trained using heterogeneous affinity-related endpoints^25^, the predicted values should not be interpreted as direct estimates for a specific biochemical assay. The present analysis also cannot distinguish how much of the observed compression arises from model calibration, training-label heterogeneity, or variation among experimental measurements. Future studies incorporating complementary datasets, such as thermodynamic binding-affinity measurements based on *K*_d_ or *K*_i_ values, may help further clarify the relationship between Boltz-2 predictions and experimental measures of molecular recognition.

In conclusion, our findings provide practical guidance for interpreting Boltz-2 predictions in the kinase drug-discovery systems examined here. Predicted log_10_(IC_50_) values were most informative for evaluating relative potency rankings rather than absolute potency values, with systematic compression toward intermediate potency estimates. Binding probability showed good binder/non-binder classification performance within the present benchmark and retained a qualitative association with potency, although its classification information substantially overlapped with that of predicted log_10_(IC_50_). Structural accuracy and potency prediction performance were not always aligned, indicating that caution is required when using structural metrics alone to assess prediction quality. The sequence-context analyses further suggest that binding-probability predictions may incorporate structural information beyond the kinase domain. When interpreted within these constraints, the present results suggest potential utility of Boltz-2 for prioritizing kinase-inhibitor relationships and exploring pharmacologically relevant interaction patterns within the systems examined. Protein kinases represent one of the most extensively characterized and pharmacologically challenging target classes. The preservation of relative interaction patterns observed across the kinase systems examined warrants further evaluation in additional protein families. Direct benchmarking will be required to determine whether the interpretive principles identified here extend beyond kinase drug-discovery research.

## Methods

### Study design

This study was designed to evaluate the performance of Boltz-2 across eight human kinases, nine clinically used inhibitors, and representative resistance mutations. Analyses focused on four aspects: (i) the relationship between predicted and experimental potency, (ii) classification of binder/non-binder, (iii) effects of protein sequence context on prediction outputs, and (iv) structural features associated with predictive performance. In addition, mutation-dependent changes in inhibitor sensitivity and variability across replicate predictions were analyzed to assess prediction robustness and reproducibility.

### Benchmark construction and experimental data curation

A benchmark panel of clinically relevant human kinases was assembled, including ABL1, SRC, EGFR, ERBB2 (HER2), KIT, PDGFRA, RIPK2, and BRAF^1–14^. Representative mutant variants associated with drug resistance or oncogenic activation were included: ABL1 Y253F and T315I, EGFR L858R, L858R/T790M, and L858R/T790M/C797S, and BRAF V600E^7,14–16^.

A diverse set of kinase inhibitors was selected to span multiple binding modes and therapeutic classes, including Imatinib, Dasatinib, Ponatinib, Gefitinib, Lapatinib, Osimertinib, Staurosporine, Sorafenib, and Dabrafenib^5–7,12^.

Experimental activity data were curated from BindingDB^41^ and PubChem BioAssay^42^. Only biochemical IC_50_ measurements for human targets were retained. Cellular assays, non-human orthologs, and records lacking interpretable IC_50_ values were excluded. When multiple records corresponded to the same original experiment, only a single representative measurement was retained. For each kinase-inhibitor pair, at least three independent measurements were required, and the median IC_50_ value was used as the representative activity.

IC_50_ values were converted to μM and expressed as log_10_(IC_50_). To assess the provenance of the experimental affinity data and potential overlap with public resources used for Boltz-2 affinity training, we performed a record-level audit for all kinase-inhibitor pairs supported by at least 10 curated IC_50_ measurements. Seven pairs met this criterion: ABL1-Imatinib, ABL1-Dasatinib, ABL1-Ponatinib, SRC-Dasatinib, EGFR-Gefitinib, EGFR-Osimertinib, and HER2-Lapatinib. For each contributing measurement, the corresponding PubChem or BindingDB record, IC_50_ value, date recorded in BindingDB when available, BindingDB DOI, and available ChEMBL assay/source identifier were collected. These records are summarized in Supplementary Table S14. Because the Boltz-2 publication does not provide a measurement-level manifest or an explicit temporal cutoff for its affinity-training dataset, this audit was used to assess potential prior availability rather than to establish actual inclusion of individual measurements in model training.

### Protein and ligand preparation

Protein sequences were obtained from UniProt canonical human entries^43^. Sequence variants were generated by introducing the corresponding amino-acid substitutions into the wild-type sequences. Three input configurations were considered: kinase domain (KD), kinase domains with flanking regions [(+)-KD-(+)], and full-length protein sequences. For EGFR dimer analyses, duplicated sequence-extended kinase constructs were provided as multimeric inputs to Boltz-2 to evaluate higher-order structural organization.

Small-molecule ligands were provided to Boltz-2 using Protein Data Bank Chemical Component Dictionary identifiers. Ligand identity was kept consistent across all prediction runs and input configurations.

### Boltz-2 prediction workflow

All kinase-inhibitor systems were evaluated using a standardized Boltz-2 workflow^25^, wherein recycling steps and diffusion samples were set to 10 and 5, respectively, while all other parameters were kept at their default values. The use_potentials option was enabled to incorporate physics-inspired interaction potentials during structure generation. For each protein-ligand pair and each input configuration, ten independent prediction runs were performed under identical conditions. Predicted log_10_(IC_50_) values and binding probabilities were recorded for all runs. For analyses requiring a representative prediction, the run whose predicted log_10_(IC_50_) value was closest to the median across replicates was selected. This strategy was used to reduce the influence of stochastic outlier predictions while retaining a representative structure from the prediction ensemble.

### AlphaFold 3 comparison workflow

AlphaFold 3 (version 3.0.1)^20^ was run locally in a Docker container using GPU acceleration. For each kinase-inhibitor complex, the same kinase-domain sequence used for the corresponding Boltz-2 prediction was supplied as a single protein chain, and the ligand was specified using its Protein Data Bank Chemical Component Dictionary identifier. Precomputed unpaired multiple-sequence alignments were supplied in the AlphaFold 3 input JSON files, whereas paired MSAs and structural templates were not used. Ten model seeds (1–10) were specified for each complex. The AlphaFold 3 data pipeline was disabled, and inference was performed using default settings for parameters not otherwise specified. Five diffusion samples were generated for each model seed, yielding 50 predicted structures per complex. The comparison was restricted to 12 wild-type kinase-inhibitor complexes for which an experimental co-crystal structure of the same wild-type kinase and the same ligand was available (Supplementary Table S12).

### Chai-1 comparison workflow

Chai-1 predictions were generated using the Chai Discovery Web application^23^. For each of the 12 wild-type kinase-inhibitor complexes included in the model comparison (Supplementary Table S12), the same kinase-domain sequence used for the corresponding Boltz-2 and AlphaFold 3 predictions was provided, and the ligand was specified using its SMILES representation. The “Use MSAs” option was enabled, and multiple-sequence alignments were generated by the server using MMseqs2. Structural templates and user-specified restraints were not used. One Web-server submission was performed for each complex, yielding five candidate structures.

### Affinity baseline calculations

To compare Boltz-2 with established affinity-scoring approaches, RTMScore^44^, MMGBSA, and MMPBSA calculations were performed for the same 57 wild-type kinase-inhibitor pairs. All three baseline calculations used the kinase-domain complex structures predicted by Boltz-2 as structural inputs and therefore represent rescoring comparisons conditioned on the same predicted complexes rather than fully independent structure-and-affinity prediction pipelines.

For RTMScore, each predicted kinase-inhibitor complex was separated into protein and ligand components. Ligand PDB files were processed using RDKit^45^ to preserve element assignments and subsequently converted to SDF format using Open Babel^46^, with bond orders inferred from the three-dimensional ligand geometry. RTMScore calculations were performed using the rtmscore_model1.pth model. The predicted ligand pose itself was used as the reference ligand to define the binding pocket, with protein residues within 10 Å of the ligand included in the pocket definition. The same ligand pose was then scored within the corresponding pocket.

MMGBSA and MMPBSA calculations were performed using the Amber24 simulation software package^47^. Ligand parameters were generated with AmberTools using the GAFF2 force field^48^. Hydrogen-containing ligand structures obtained from PubChem in SDF format were processed with Antechamber, and AM1-BCC partial charges were assigned using the net charge of each ligand. Additional ligand parameters were generated with parmchk2. Kinase-inhibitor complexes were prepared in tleap using ff19SB^49^ for the kinase and GAFF2 for the ligand. Complexes were solvated in rectangular TIP3P water boxes^50^ with a minimum solute-to-box boundary of 15 Å and neutralized with Na^+^ or Cl^−^ ions. Energy minimization and molecular-dynamics simulations were carried out using Amber24. Following 10,000 steps of unrestrained energy minimization, 5.0-ns molecular-dynamics simulations were performed at 310 K under NVT conditions using a 1.0-fs time step, a 9.0-Å nonbonded cutoff, SHAKE constraints^51^ on bonds involving hydrogen atoms, and Langevin temperature control with a collision frequency of 1.0 ps^−1^ and the particle mesh Ewald method^52^ for long-range electrostatic interactions.

MMGBSA and MMPBSA binding energies were calculated using MMPBSA.py in Amber24 from 20 snapshots sampled at 0.1-ns intervals from 3.1 to 5.0 ns of each trajectory. Entropic contributions were not included. MMGBSA calculations used igb = 5 (OBC2)^53^ and saltcon = 0.1 M, whereas MMPBSA calculations used indi = 2.0, istrng = 0.1 M, and radiopt = 0; other parameters were left at the Amber default settings. Ligand positional stability during the MMGBSA and MMPBSA sampling window was evaluated using the same 20 snapshots. For each snapshot, the kinase domain was structurally aligned to the initial Boltz-2-predicted complex, after which the heavy-atom RMSD of the ligand was calculated without separately fitting the ligand, using the initial Boltz-2 ligand pose as the reference. Mean ligand RMSD and standard deviation were calculated across the 20 snapshots.

### Structural analysis and RMSD calculation

Structural analyses were performed using predictions generated from kinase domain sequence inputs. For each Boltz-2 kinase-inhibitor pair, the representative structure was defined as the prediction whose predicted log_10_(IC_50_) value was closest to the median across ten independent Boltz-2 predictions. For AlphaFold 3, the structure with the highest ranking score among 50 predictions generated from ten model seeds and five diffusion samples per seed was selected as the representative structure. For Chai-1, the structure with the highest aggregate score among the five candidate structures generated in a single Web-server submission was selected as the representative structure.

Predicted structures were aligned to experimental structures using kinase-domain Cα atoms. Structural accuracy was quantified using two metrics: RMSD_KD_, defined as the Cα RMSD of the kinase domain, and RMSD_Lig_, defined as the heavy-atom RMSD of the ligand after kinase-domain alignment. The same alignment and RMSD calculation procedures were applied to the representative AlphaFold 3 and Chai-1 models used for comparison.

Reference experimental structures and corresponding PDB identifiers are listed in Supplementary Table S11. Structural analyses and visualization were performed using PyMOL^54^.

Kinase conformational states were classified based on αC-helix positioning and DFG motif orientation. αC-IN states were defined by the presence of the conserved β3 Lys-αC Glu salt bridge, with a distance of ≤ 4 Å between the NZ atom of the β3 lysine residue and the CD atom of the αC-helix glutamate residue. Distances greater than 4 Å were classified as αC-OUT. DFG motif conformations were classified using the distance between the CD atom of the αC-helix glutamate residue and the Cα atom of the DFG phenylalanine residue. Distances ≤5 Å were classified as DFG-IN, whereas distances >5 Å were classified as DFG-OUT. Distance thresholds were selected based on inspection of representative kinase crystal structures exhibiting canonical active and inactive conformations.

To assess structural variability, alternative ligand binding poses and RMSD distributions were analyzed across ten independent predictions for selected systems.

### Time-resolved structural overlap assessment

To assess potential structural training overlap among the complexes used for ligand-pose comparison, we surveyed the PDB for experimental structures containing the same wild-type human kinase and the same inhibitor as each of the 12 benchmark complexes. An exact complex was defined as a publicly available experimental structure containing the same wild-type human kinase and the same inhibitor as the corresponding benchmark pair. Structures containing mutant or non-human kinases, oncogenic fusion proteins rather than the corresponding wild-type kinase, different protein targets, or different ligands were excluded. For each exact complex, the PDB release date was recorded, and structures released before the stated Boltz-2 structural training cutoff of 1 June 2023 were classified as pre-cutoff exact complexes. The number and identity of pre-cutoff exact complexes were determined for each kinase-inhibitor pair. The identified PDB entries and corresponding ligand-pose RMSD values are summarized in Supplementary Table S13. This analysis identifies potential structural training exposure based on public availability before the stated cutoff, but does not establish whether individual structures were actually included in the Boltz-2 training data.

### Ligand conformational flexibility analysis

Ligand conformational flexibility was quantified using an ensemble-based RMSD descriptor (FI_RMSD). Three-dimensional conformers were generated using the ETKDG algorithm implemented in RDKit^45^. Up to 30 conformers per ligand were sampled and energy-minimized using the MMFF94 force field, or UFF when parameters were unavailable.

All conformers were aligned by heavy-atom least-squares superposition, and pairwise RMSD values were calculated. The flexibility index was defined as the average pairwise RMSD across the conformer ensemble as follows:

1 $${FI}{{\rm{\_}}}{RMSD}=\frac{2}{N(N-1)}\sum {{RMSD}}_{i,j}$$

where *N* is the number of conformers.

This metric was used to compare intrinsic ligand flexibility across the inhibitors included in the benchmark.

### Statistical analysis

Classification performance was evaluated by categorizing kinase–inhibitor pairs into binder and non-binder classes, where pairs with experimental IC50 values below 1 μM (log_10_(IC_50_) < 0) were defined as binders. Receiver-operating characteristic (ROC) analysis was performed to evaluate classification performance. The optimal classification threshold was determined using Youden’s J statistic.

Associations between predicted and experimental log_10_(IC_50_) values were evaluated using correlation analyses. Associations between predicted binding probability and predicted log_10_(IC_50_) were evaluated using Pearson correlation separately for each sequence input definition. Ordinary least-squares linear regression was used to quantify calibration between predicted and experimental log_10_(IC_50_) values, with predicted values treated as the dependent variable and experimental values as the independent variable. To assess the uncertainty of the overall potency associations, pair-level bootstrap analyses were performed using the 57 wild-type kinase-inhibitor pairs as the resampling unit. For each sequence input definition, 20,000 bootstrap samples were generated by sampling kinase-inhibitor pairs with replacement. Pearson correlation coefficients, Spearman rank correlation coefficients, and ordinary least-squares regression slopes were recalculated for each bootstrap sample, and percentile-based 95% confidence intervals were obtained from the resulting bootstrap distributions. For the binding-probability ROC analyses, ROC AUC values and optimal thresholds maximizing Youden’s J statistic were also recalculated for each bootstrap sample, and percentile-based 95% confidence intervals were obtained from the corresponding bootstrap distributions. The dispersion of the predicted and experimental distributions was compared using standard deviations and interquartile ranges.

To determine whether the predictive agreement of Boltz-2 could be explained by simple kinase- or inhibitor-specific potency patterns, four statistical baselines were evaluated using leave-one-pair-out cross-validation. In each fold, one kinase-inhibitor pair was held out, and baseline predictions were calculated using only the remaining experimental log_10_(IC_50_) values. The global-mean baseline assigned the mean experimental log_10_(IC_50_) of the training data to the held-out pair. The kinase-mean baseline used the mean value for the same kinase across the remaining inhibitors, whereas the inhibitor-mean baseline used the mean value for the same inhibitor across the remaining kinases. A two-way additive model was fitted to the training data using kinase and inhibitor identities as categorical effects, without an explicit kinase-inhibitor interaction term. Baseline and Boltz-2 predictions were compared using Pearson correlation, Spearman rank correlation, root-mean-square error, mean absolute error, and the coefficient of determination.

To compare the classification information provided by predicted log_10_(IC_50_), binding probability, and their combination, leave-one-kinase-out cross-validation was performed separately for each sequence input definition. In each fold, all kinase-inhibitor pairs associated with one kinase were held out for testing, and the remaining kinases were used for model fitting. Logistic-regression classifiers were constructed using predicted log_10_(IC_50_) alone, binding probability alone, or both outputs as input features. Input features were standardized using parameters estimated from the training data within each fold, and the same transformation was applied to the held-out kinase. Predicted class probabilities were converted to binary classifications using a threshold of 0.5. Performance was evaluated from predictions pooled across all held-out folds using ROC AUC, balanced accuracy, sensitivity, and specificity.

Residuals were defined as predicted minus experimental log_10_(IC_50_). Associations between residuals and experimental log_10_(IC_50_) values were assessed using Pearson correlation. For potency-stratified analyses, kinase-inhibitor pairs with experimental log_10_(IC_50_) values below −1 were classified as strong binders, whereas pairs with values of 0 or greater were classified as weak binders or non-binders. Mean residuals were calculated separately for these groups to assess potency-dependent prediction bias.

Relationships between RMSD_Lig_ and residuals were analyzed using Pearson correlation and group-wise comparisons based on predefined RMSD thresholds.

Group comparisons were performed using the Mann–Whitney U test, and effect sizes were quantified using Cliff’s *δ*.

For affinity baseline comparisons, Pearson and Spearman correlation coefficients between each scoring method and experimental log_10_(IC_50_) values were calculated across all 57 wild-type kinase-inhibitor pairs. Correlations were also calculated separately for each kinase represented by multiple wild-type inhibitor pairs; RIPK2 was excluded from the kinase-wise analysis because only one wild-type inhibitor pair was available. Because higher RTMScore values indicate stronger predicted binding whereas lower experimental log_10_(IC_50_) values indicate stronger activity, favorable agreement for RTMScore is reflected by negative correlation coefficients.

All statistical analyses were performed using Python-based scientific computing libraries, including NumPy, SciPy, and scikit-learn.

## Supplementary information

Supplementary Information

## Data Availability

The curated biochemical IC50 dataset, Boltz-2 prediction outputs, and all processed data used for the analyses in this study are provided in the Supplementary Information. Source databases used for data curation are publicly available from BindingDB and PubChem BioAssay.

## Code availability

The custom scripts used for data processing, statistical analysis, and figure generation in this study are not publicly available but may be made available to qualified researchers on reasonable request from the corresponding author.
