# Supplementary material for: Benchmarking Boltz-2 for relative kinase-inhibitor interaction patterns across conformational states and resistance mutations
Source: NPJ Drug Discov. 2026 Oct 12;3:46. doi: 10.1038/s44386-026-00086-x (PMC13658129; doi:10.1038/s44386-026-00086-x)
Supplement: Supplementary file 1 [file 44386_2026_86_MOESM1_ESM.docx]

**SUPPORTING INFORMATION**

**Benchmarking Boltz-2 for relative kinase-inhibitor interaction patterns across conformational states and resistance mutations**

Atsushi Suenaga^1,*^, Noriaki Okimoto^2,3^

^1^ Department of Informatics and Data Science, Sanyo-Onoda City University, 1-1-1 Daigakudori, Sanyo-Onoda, Yamaguchi 756-0884, Japan

^2^ Laboratory for Computational Molecular Design, RIKEN Center for Biosystems Dynamics Research (BDR), 6-7-1 Minatojima minami-machi, Chuou-ku, Kobe, Hyogo, 650-0047, Japan

^3^ Drug Discovery Molecular Simulation Platform Unit, RIKEN Center for Biosystems Dynamics Research (BDR), 6-7-1 Minatojima minami-machi, Chuou-ku, Kobe, Hyogo, 650-0047, Japan

*To whom correspondence should be addressed: email: asuenaga@rs.socu.ac.jp

**Supplementary Figures**

Fig. S1 | Domain organization and sequence input definitions used for Boltz‑2 predictions.

Fig. S2 | Residual analysis of Boltz-2 potency predictions across sequence input definitions.

Fig. S3 | Prediction errors across kinase-inhibitor combinations under different sequence input definitions.

Fig. S4 | Correlation of ligand conformational flexibility with inhibitor‑specific prediction accuracy.

Fig. S5 | ROC analysis for classification of binders versus non-binders using predicted binding probability.

Fig. S6 | Structural comparison of additional kinase-inhibitor complexes not shown in Fig. 4.

Fig. S7 | Global structural comparison of kinase domains between experimental and predicted models.

Fig. S8 | Variability of predicted structures across independent Boltz‑2 runs for EGFR-Lapatinib.

Fig. S9 | Sequence-context-dependent structural organization in additional kinase-inhibitor systems.

Fig. S10 | Comparison of predicted and experimental EGFR dimer organization using sequence-extended inputs.

**Supplementary Tables**

Table S1 | Curated experimental IC₅₀ benchmark dataset for wild-type kinase-inhibitor pairs.

Table S2 | Pair-level bootstrap confidence intervals for potency associations, regression slopes, and binder/non-binder classification metrics across sequence input definitions.

Table S3 | Comparison of Boltz-2 potency predictions with simple statistical baselines.

Table S4 | Complete Boltz-2 prediction outputs for wild-type systems.

Table S5 | Pearson correlation coefficients between experimental and predicted log_10_(IC_50_) values stratified by kinase and inhibitor.

Table S6 | Leave-one-kinase-out comparison of binder/non-binder classification using Boltz-2 potency and binding-probability outputs.

Table S7 | Pearson correlation coefficients between experimental log_10_(IC_50_) values and predicted binding probability scores stratified by kinase and inhibitor.

Table S8 | Curated experimental IC₅₀ benchmark dataset for mutant kinase-inhibitor pairs.

Table S9 | Complete Boltz-2 prediction outputs for mutant systems.

Table S10 | Conformational states of the αC-helix and DFG motif in predicted kinase-inhibitor complex structures.

Table S11 | Structural validation metrics for predicted kinase-inhibitor complex models against reference experimental structures.

Table S12 | Comparison of ligand-pose accuracy between Boltz-2, AlphaFold 3, and Chai-1 for wild-type kinase-inhibitor complexes.

Table S13 | Time-resolved structural overlap audit of exact wild-type kinase-inhibitor complexes relative to the Boltz-2 structural training cutoff, with ligand-pose accuracy of Boltz-2, AlphaFold 3, and Chai-1.

Table S14 | Record-level provenance of affinity measurements for the seven most extensively characterized kinase-inhibitor pairs.

Table S15 | Comparison of experimental potency, Boltz-2 predictions, and established affinity-scoring baselines across 57 wild-type kinase-inhibitor pairs.

Table S16 | Global correlations between predicted affinity scores and experimental log_10_(IC_50_) values.

Table S17 | Kinase-wise correlations between affinity scores and experimental log_10_(IC_50_) values.

Table S18 | Effects of sequence-context extension on predicted binding probability and kinase conformational states in EGFR inhibitor systems.

**Fig. S1 | Domain organization and sequence input definitions used for Boltz‑2 predictions.** Schematic representations of the domain architecture and sequence boundaries used for kinase domain only (KD), kinase domain with flanking regions [(+)‑KD‑(+)], and full‑length input definitions. Red segments denote kinase domains, blue segments indicate predicted intrinsically disordered regions, and green segments represent annotated regulatory regions involved in dimerization, phosphorylation, or activation. Construct boundaries for each sequence input definition are indicated for the kinases examined in this study.

**Fig. S2 | Residual analysis of Boltz-2 potency predictions across sequence input definitions.** Residuals were defined as predicted minus experimental log_10_(IC_50_). Scatter plots show residuals versus experimental log_10_(IC_50_) values for 57 kinase-inhibitor pairs using (A) kinase domain only, (B) kinase domain + flanking regions, and (C) full-length protein sequences as model inputs. Colors denote kinase targets and symbol shapes denote inhibitors. Solid red lines indicate linear regression fits with shaded 95% confidence intervals, and dashed horizontal lines indicate zero residual. Pearson correlation coefficients (*r*) are reported for each input definition.

**Fig. S3 | Prediction errors across kinase-inhibitor combinations under different sequence‑input definitions.** Heatmaps show squared prediction errors, defined as [Predicted log_10_(IC_50_) − Experimental log_10_(IC_50_)]^2^, for each kinase-inhibitor pair under three sequence‑input definitions: (A) kinase domain only, (B) kinase domain with flanking regions, and (C) full‑length protein sequences. Lower values (blue) indicate higher predictive accuracy, whereas higher values (red) indicate larger prediction errors. Blank cells indicate unavailable benchmark measurements.

**Fig. S4 | Correlation of ligand conformational flexibility with inhibitor‑specific prediction accuracy.** (A) Relationship between ligand conformational flexibility (FI_RMSD) and inhibitor‑wise Pearson correlation coefficients between experimental and predicted log_10_(IC_50_) values. (B) Relationship between FI_RMSD and inhibitor‑wise mean squared prediction error for log_10_(IC_50_) values. Each point represents one inhibitor.

**Fig. S5 | ROC analysis for classification of binders versus non-binders using predicted binding probability.** Receiver operating characteristic curves were generated using predicted binding‑probability scores from three sequence‑input definitions: (A) kinase domain only, (B) kinase domain with flanking regions, and (C) full‑length proteins. Experimental activity labels were assigned using a threshold of IC_50_ < 1 µM (log_10_(IC_50_) < 0) for binders and IC_50_ ≥ 1 µM for non‑binders. Optimal classification thresholds were selected by maximizing Youden’s index. AUC values and selected thresholds are indicated in each panel. These optimal thresholds were used for the classification analyses shown in Fig. 2A-C.

**Fig. S6 | Structural comparison of additional kinase-inhibitor complexes not shown in Fig. 4.** Kinase domains are shown as cartoons and inhibitors as sticks. Experimental structures are colored green for kinases and orange for ligands, whereas predicted structures are colored red for kinases and cyan for ligands. The αC‑helix and DFG motif are highlighted for reference.

**Fig. S7 | Global structural comparison of kinase domains between experimental and predicted models.** Superposition of experimental and predicted kinase domain structures for all kinase-inhibitor complexes with available crystal structures. Experimental structures are shown in green and predicted structures in red. Structures are aligned using the kinase domain. Each panel corresponds to one kinase-inhibitor pair.

**Fig. S8 | Variability of predicted structures across independent Boltz‑2 runs for EGFR-Lapatinib.** Representative predicted structures and outlier poses from independent Boltz-2 runs are shown for two sequence‑input definitions: kinase domain only (KD, left) and the sequence‑extended (+)‑KD‑(+) definition (right). Experimental structures are shown in green, and representative predicted structures (i.e., poses whose predicted log_10_(IC_50_) values were closest to the median across the ten independent predictions) are shown in red. Outlier structures are shown in blue, corresponding to the pose with the highest RMSD_Lig_ for KD (Pose No. 4) and the pose with the lowest predicted log_10_(IC_50_) for (+)‑KD‑(+) (Pose No. 10). Tables summarize RMSD_KD_, RMSD_Lig_, predicted log_10_(IC_50_), and binding probability (BP) for the selected poses.

**Fig. S9 | Sequence-context-dependent structural organization in additional kinase-inhibitor systems.** Predicted structures of HER2-Lapatinib (ErbB family kinase showing stronger sequence-context effects), ABL1-Dasatinib (representative non-ErbB tyrosine kinase system showing limited sequence-context effects), and BRAF-Sorafenib (serine/threonine kinase system showing limited sequence-context effects) complexes generated using the (+)-KD-(+) sequence definition. Upper panels show surface representations and lower panels show cartoon representations of the predicted complexes. Green indicates the kinase domain, red indicates the αC-helix, black indicates the DFG motif, magenta indicates the activation loop, and yellow indicates the bound inhibitor. Blue and cyan represent additional sequence regions located N-terminal and C-terminal to the kinase domain, respectively.

**Fig. S10 | Comparison of predicted and experimental EGFR dimer organization using sequence-extended inputs.** Predicted EGFR dimer structures generated using the (+)-KD-(+) sequence definition are compared with an experimentally determined EGFR dimer structure. Upper panels show cartoon representations and lower panels show surface representations. In the predicted model, monomer 1 and monomer 2 kinase domains are colored green and wheat, respectively. N-terminal extended regions are colored blue, whereas C-terminal extended regions are colored cyan for monomer 1 and gray for monomer 2. The αC-helices are colored red and the activation loops are colored magenta. Bound inhibitors are shown as yellow sticks. The predicted model contains Gefitinib, whereas the experimental structure corresponds to PDB entry 9P9U (ligand CCD: 6JS).

**Table S1. Curated experimental IC₅₀ benchmark dataset for wild-type kinase-inhibitor pairs.** Representative IC_50_ values were obtained as the median of publicly available biochemical measurements curated from BindingDB and PubChem BioAssay. IC_50_ values are reported in µM, and log_10_(IC_50_) values were calculated using the µM scale. Duplicate entries were removed. The column *n* indicates the number of independent experimental IC_50_ measurements; only kinase-inhibitor pairs with *n* ≥ 3 were included in the benchmark dataset.

| **Kinase** | **Inhibitor** | ***n*** | **Median IC_50_** | **log_10_(IC_50_)** |
| --- | --- | --- | --- | --- |
| ABL1 | Imatinib | 10 | 0.10 | -1.00 |
|  | Dasatinib | 12 | 0.0015 | -2.82 |
|  | Ponatinib | 13 | 0.0005 | -3.30 |
|  | Gefitinib | 3 | 8.50 | 0.93 |
|  | Lapatinib | 3 | 9.20 | 0.96 |
|  | Osimertinib | 3 | 7.80 | 0.89 |
|  | Staurosporine | 7 | 0.006 | -2.22 |
|  | Sorafenib | 3 | 0.43 | -0.37 |
| PDGFRA | Imatinib | 6 | 0.08 | -1.10 |
|  | Dasatinib | 4 | 0.045 | -1.35 |
|  | Ponatinib | 5 | 0.012 | -1.92 |
|  | Gefitinib | 3 | 5.20 | 0.72 |
|  | Lapatinib | 3 | 6.80 | 0.83 |
|  | Osimertinib | 3 | 6.10 | 0.79 |
|  | Staurosporine | 4 | 0.0045 | -2.35 |
|  | Sorafenib | 3 | 0.10 | -1.00 |
| KIT | Imatinib | 9 | 0.09 | -1.05 |
|  | Dasatinib | 7 | 0.018 | -1.75 |
|  | Ponatinib | 7 | 0.01 | -2.00 |
|  | Gefitinib | 3 | 3.80 | 0.58 |
|  | Lapatinib | 3 | 5.50 | 0.74 |
|  | Osimertinib | 3 | 5.40 | 0.73 |
|  | Staurosporine | 5 | 0.0038 | -2.42 |
|  | Sorafenib | 7 | 0.07 | -1.16 |
| SRC | Imatinib | 3 | 7.20 | 0.86 |
|  | Dasatinib | 10 | 0.0008 | -3.10 |
|  | Ponatinib | 8 | 0.004 | -2.40 |
|  | Gefitinib | 4 | 6.10 | 0.79 |
|  | Lapatinib | 3 | 7.40 | 0.87 |
|  | Osimertinib | 3 | 8.20 | 0.91 |
|  | Staurosporine | 8 | 0.0012 | -2.92 |
|  | Sorafenib | 3 | 0.55 | -0.26 |
| EGFR | Imatinib | 3 | 12.00 | 1.08 |
|  | Dasatinib | 3 | 1.80 | 0.26 |
|  | Ponatinib | 3 | 0.85 | -0.07 |
|  | Gefitinib | 12 | 0.033 | -1.48 |
|  | Lapatinib | 9 | 0.11 | -0.96 |
|  | Osimertinib | 10 | 0.045 | -1.35 |
|  | Staurosporine | 6 | 0.012 | -1.92 |
|  | Sorafenib | 3 | 3.20 | 0.51 |
| HER2 | Imatinib | 3 | 9.50 | 0.98 |
|  | Dasatinib | 3 | 0.95 | -0.02 |
|  | Ponatinib | 3 | 0.42 | -0.38 |
|  | Gefitinib | 5 | 0.38 | -0.42 |
|  | Lapatinib | 10 | 0.018 | -1.75 |
|  | Osimertinib | 4 | 0.52 | -0.28 |
|  | Staurosporine | 4 | 0.008 | -2.10 |
| RIPK2 | Staurosporine | 3 | 0.022 | -1.66 |
| BRAF | Imatinib | 3 | 15.00 | 1.18 |
|  | Dasatinib | 3 | 7.50 | 0.88 |
|  | Ponatinib | 3 | 1.80 | 0.26 |
|  | Gefitinib | 3 | 12.00 | 1.08 |
|  | Lapatinib | 3 | 14.00 | 1.15 |
|  | Osimertinib | 3 | 10.50 | 1.02 |
|  | Staurosporine | 5 | 0.028 | -1.55 |
|  | Sorafenib | 3 | 0.028 | -1.55 |
|  | Dabrafenib | 6 | 0.0032 | -2.50 |

**Table S2. Pair-level bootstrap confidence intervals for potency associations, regression slopes, and binder/non-binder classification metrics across sequence input definitions.** Pair-level bootstrap analyses were performed using the 57 wild-type kinase-inhibitor pairs as the resampling unit. For each sequence input definition, 20,000 bootstrap samples were generated with replacement, and percentile-based 95% confidence intervals were calculated for Pearson correlation coefficients, Spearman rank correlation coefficients, ordinary least-squares regression slopes, ROC AUC values, and optimal binding-probability thresholds (reported as “Threshold” in the table). Binders were defined as kinase-inhibitor pairs with experimental log_10_(IC_50_) < 0, and the optimal threshold was defined as the binding-probability cutoff that maximized the Youden index.

| **Region** | **Pearson *r*** | **Bootstrap 95% CI** | **Spearman *ρ*** | **Bootstrap 95% CI** | **Slope** | **Bootstrap 95% CI** | **ROC AUC** | **Bootstrap 95% CI** | **Threshold** | **Bootstrap 95% CI** |
| --- | --- | --- | --- | --- | --- | --- | --- | --- | --- | --- |
| KD | 0.821 | 0.745–0.881 | 0.817 | 0.719–0.872 | 0.580 | 0.481–0.684 | 0.886 | 0.784–0.962 | 0.29 | 0.25–0.70 |
| (+)-KD-(+) | 0.812 | 0.722–0.880 | 0.805 | 0.691–0.867 | 0.578 | 0.472–0.682 | 0.916 | 0.824–0.981 | 0.59 | 0.28–0.72 |
| Full-length | 0.806 | 0.725–0.871 | 0.797 | 0.689–0.861 | 0.562 | 0.458–0.670 | 0.918 | 0.825–0.987 | 0.50 | 0.37–0.70 |

**Table S3. Comparison of Boltz-2 potency predictions with simple statistical baselines.** Four statistical baselines were evaluated using leave-one-pair-out cross-validation to determine whether the agreement between predicted and experimental log_10_(IC_50_) values could be explained by global, kinase-specific, or inhibitor-specific potency patterns. The additive model incorporated kinase and inhibitor effects without an explicit kinase-inhibitor interaction term. Baseline and Boltz-2 predictions were compared using Pearson correlation, Spearman rank correlation, root-mean-square error (RMSE), mean absolute error (MAE), and the coefficient of determination (*R*^2^).

| **Model** | **Pearson *r*** | **Spearman *ρ*** | **RMSE** | **MAE** | ***R*^2^** |
| --- | --- | --- | --- | --- | --- |
| Global mean | — | — | 1.381 | 1.242 | −0.036 |
| Kinase mean | −0.392 | −0.398 | 1.516 | 1.346 | −0.248 |
| Inhibitor mean | 0.531 | 0.419 | 1.168 | 0.991 | 0.260 |
| Additive kinase + inhibitor | 0.466 | 0.473 | 1.272 | 1.052 | 0.122 |
| Boltz-2: KD | 0.821 | 0.817 | 0.799 | 0.646 | 0.654 |
| Boltz-2: (+)-KD-(+) | 0.812 | 0.805 | 0.822 | 0.670 | 0.633 |
| Boltz-2: Full-length | 0.806 | 0.797 | 0.834 | 0.686 | 0.623 |

**Table S4. Complete Boltz-2 prediction outputs for wild-type systems.** Predicted log_10_(IC_50_) values and binding‑probability (BP) scores are reported for each kinase-inhibitor pair across multiple sequence‑input definitions, including the kinase domain (KD), the sequence‑extended (+)‑KD‑(+), and full‑length protein inputs. Experimental log_10_(IC_50_) values are shown for comparison. Predicted values are reported as the median across ten independent Boltz-2 runs, with standard deviations shown in parentheses.

| **Kinase** | **Inhibitor** | **Region** | **Exp Log_10_(IC_50_)** | **Pred Log_10_(IC_50_)** | **BP** |
| --- | --- | --- | --- | --- | --- |
| ABL1 | Imatinib | KD | -1.00 | -0.02 (0.05) | 0.37 (0.01) |
| ABL1 | Imatinib | (+)-KD-(+) | -1.00 | -0.47 (0.05) | 0.43 (0.01) |
| ABL1 | Imatinib | Full ABL1 | -1.00 | -0.71 (0.24) | 0.59 (0.05) |
| ABL1 | Imatinib | Full BCR-ABL1 | -1.00 | -0.50 (0.30) | 0.49 (0.11) |
| ABL1 | Dasatinib | KD | -2.82 | -1.75 (0.07) | 0.88 (0.01) |
| ABL1 | Dasatinib | (+)-KD-(+) | -2.82 | -2.21 (0.04) | 0.94 (0.01) |
| ABL1 | Dasatinib | Full ABL1 | -2.82 | -2.23 (0.16) | 0.95 (0.01) |
| ABL1 | Dasatinib | Full BCR-ABL1 | -2.82 | -2.20 (0.24) | 0.95 (0.02) |
| ABL1 | Ponatinib | KD | -3.30 | -1.76 (0.05) | 0.84 (0.01) |
| ABL1 | Ponatinib | (+)-KD-(+) | -3.30 | -2.11 (0.07) | 0.88 (0.11) |
| ABL1 | Ponatinib | Full ABL1 | -3.30 | -1.84 (0.13) | 0.89 (0.02) |
| ABL1 | Ponatinib | Full BCR-ABL1 | -3.30 | -1.94 (0.15) | 0.88 (0.03) |
| ABL1 | Gefitinib | KD | 0.93 | 1.02 (0.09) | 0.14 (0.01) |
| ABL1 | Gefitinib | (+)-KD-(+) | 0.93 | 0.53 (0.07) | 0.20 (0.02) |
| ABL1 | Gefitinib | Full ABL1 | 0.93 | 0.76 (0.15) | 0.35 (0.13) |
| ABL1 | Gefitinib | Full BCR-ABL1 | 0.93 | 0.72 (0.15) | 0.46 (0.16) |
| ABL1 | Lapatinib | KD | 0.96 | 0.10 (0.14) | 0.05 (0.01) |
| ABL1 | Lapatinib | (+)-KD-(+) | 0.96 | 0.18 (0.14) | 0.06 (0.01) |
| ABL1 | Lapatinib | Full ABL1 | 0.96 | -0.13 (0.26) | 0.10 (0.03) |
| ABL1 | Lapatinib | Full BCR-ABL1 | 0.96 | -0.07 (0.15) | 0.09 (0.03) |
| ABL1 | Osimertinib | KD | 0.89 | 0.25 (0.09) | 0.25 (0.21) |
| ABL1 | Osimertinib | (+)-KD-(+) | 0.89 | 0.47 (0.09) | 0.23 (0.01) |
| ABL1 | Osimertinib | Full ABL1 | 0.89 | -0.01 (0.51) | 0.49 (0.18) |
| ABL1 | Osimertinib | Full BCR-ABL1 | 0.89 | 0.06 (0.44) | 0.48 (0.10) |
| ABL1 | Staurosporine | KD | -2.22 | -0.73 (0.39) | 0.26 (0.02) |
| ABL1 | Staurosporine | (+)-KD-(+) | -2.22 | -0.73 (0.02) | 0.33 (0.02) |
| ABL1 | Staurosporine | Full ABL1 | -2.22 | -0.84 (0.22) | 0.74 (0.11) |
| ABL1 | Staurosporine | Full BCR-ABL1 | -2.22 | -0.89 (0.17) | 0.79 (0.12) |
| ABL1 | Sorafenib | KD | -0.37 | -0.45 (0.18) | 0.54 (0.09) |
| ABL1 | Sorafenib | (+)-KD-(+) | -0.37 | -1.02 (0.04) | 0.72 (0.04) |
| ABL1 | Sorafenib | Full ABL1 | -0.37 | -0.72 (0.19) | 0.73 (0.05) |
| ABL1 | Sorafenib | Full BCR-ABL1 | -0.37 | -0.82 (0.17) | 0.72 (0.04) |
| PDGFRA | Imatinib | KD | -1.10 | -1.06 (0.12) | 0.76 (0.03) |
| PDGFRA | Imatinib | (+)-KD-(+) | -1.10 | -1.23 (0.15) | 0.79 (0.04) |
| PDGFRA | Imatinib | Full PDGFRA | -1.10 | -0.77 (0.18) | 0.72 (0.05) |
| PDGFRA | Dasatinib | KD | -1.35 | -2.28 (0.12) | 0.95 (0.01) |
| PDGFRA | Dasatinib | (+)-KD-(+) | -1.35 | -2.30 (0.23) | 0.98 (0.02) |
| PDGFRA | Dasatinib | Full PDGFRA | -1.35 | -2.29 (0.21) | 0.97 (0.01) |
| PDGFRA | Ponatinib | KD | -1.92 | -2.27 (0.10) | 0.91 (0.02) |
| PDGFRA | Ponatinib | (+)-KD-(+) | -1.92 | -2.15 (0.08) | 0.91 (0.03) |
| PDGFRA | Ponatinib | Full PDGFRA | -1.92 | -2.13 (0.13) | 0.95 (0.02) |
| PDGFRA | Gefitinib | KD | 0.72 | 0.26 (0.17) | 0.36 (0.06) |
| PDGFRA | Gefitinib | (+)-KD-(+) | 0.72 | 0.88 (0.17) | 0.35 (0.05) |
| PDGFRA | Gefitinib | Full PDGFRA | 0.72 | 0.84 (0.18) | 0.34 (0.03) |
| PDGFRA | Lapatinib | KD | 0.83 | -0.55 (0.19) | 0.13 (0.04) |
| PDGFRA | Lapatinib | (+)-KD-(+) | 0.83 | -0.22 (0.25) | 0.14 (0.04) |
| PDGFRA | Lapatinib | Full PDGFRA | 0.83 | -0.34 (0.33) | 0.20 (0.08) |
| PDGFRA | Osimertinib | KD | 0.79 | 0.25 (0.35) | 0.21 (0.04) |
| PDGFRA | Osimertinib | (+)-KD-(+) | 0.79 | 0.13 (0.21) | 0.34 (0.03) |
| PDGFRA | Osimertinib | Full PDGFRA | 0.79 | 0.13 (0.18) | 0.33 (0.03) |
| PDGFRA | Staurosporine | KD | -2.35 | -2.58 (0.09) | 0.93 (0.02) |
| PDGFRA | Staurosporine | (+)-KD-(+) | -2.35 | -2.05 (0.06) | 0.80 (0.02) |
| PDGFRA | Staurosporine | Full PDGFRA | -2.35 | -2.04 (0.06) | 0.81 (0.01) |
| PDGFRA | Sorafenib | KD | -1.00 | -1.55 (0.11) | 0.87 (0.02) |
| PDGFRA | Sorafenib | (+)-KD-(+) | -1.00 | -1.74 (0.07) | 0.98 (0.01) |
| PDGFRA | Sorafenib | Full PDGFRA | -1.00 | -1.67 (0.08) | 0.96 (0.02) |
| KIT | Imatinib | KD | -1.05 | -0.90 (0.33) | 0.70 (0.08) |
| KIT | Imatinib | (+)-KD-(+) | -1.05 | -0.95 (0.39) | 0.61 (0.08) |
| KIT | Imatinib | Full KIT | -1.05 | -0.32 (0.28) | 0.63 (0.07) |
| KIT | Dasatinib | KD | -1.75 | -2.13 (0.23) | 0.95 (0.01) |
| KIT | Dasatinib | (+)-KD-(+) | -1.75 | -2.19 (0.17) | 0.96 (0.01) |
| KIT | Dasatinib | Full KIT | -1.75 | -1.54 (0.26) | 0.91 (0.01) |
| KIT | Ponatinib | KD | -2.00 | -2.04 (0.10) | 0.90 (0.02) |
| KIT | Ponatinib | (+)-KD-(+) | -2.00 | -2.04 (0.13) | 0.90 (0.01) |
| KIT | Ponatinib | Full KIT | -2.00 | -1.94 (0.09) | 0.89 (0.01) |
| KIT | Gefitinib | KD | 0.58 | 0.61 (0.08) | 0.39 (0.06) |
| KIT | Gefitinib | (+)-KD-(+) | 0.58 | 0.57 (0.27) | 0.47 (0.05) |
| KIT | Gefitinib | Full KIT | 0.58 | 1.20 (0.26) | 0.33 (0.05) |
| KIT | Lapatinib | KD | 0.74 | -0.20 (0.30) | 0.07 (0.02) |
| KIT | Lapatinib | (+)-KD-(+) | 0.74 | -0.06 (0.43) | 0.08 (0.02) |
| KIT | Lapatinib | Full KIT | 0.74 | 0.03 (0.21) | 0.08 (0.01) |
| KIT | Osimertinib | KD | 0.73 | 0.64 (0.47) | 0.16 (0.03) |
| KIT | Osimertinib | (+)-KD-(+) | 0.73 | -0.13 (0.20) | 0.18 (0.05) |
| KIT | Osimertinib | Full KIT | 0.73 | 0.22 (0.22) | 0.24 (0.03) |
| KIT | Staurosporine | KD | -2.42 | -2.29 (0.10) | 0.90 (0.02) |
| KIT | Staurosporine | (+)-KD-(+) | -2.42 | -2.31 (0.12) | 0.92 (0.02) |
| KIT | Staurosporine | Full KIT | -2.42 | -2.01 (0.20) | 0.83 (0.02) |
| KIT | Sorafenib | KD | -1.16 | -1.07 (0.15) | 0.80 (0.04) |
| KIT | Sorafenib | (+)-KD-(+) | -1.16 | -1.11 (0.16) | 0.86 (0.04) |
| KIT | Sorafenib | Full KIT | -1.16 | -1.24 (0.10) | 0.89 (0.02) |
| SRC | Imatinib | KD | 0.86 | 0.40 (0.05) | 0.24 (0.01) |
| SRC | Imatinib | (+)-KD-(+) | 0.86 | 0.36 (0.05) | 0.21 (0.04) |
| SRC | Imatinib | Full SRC | 0.86 | 0.35 (0.13) | 0.25 (0.02) |
| SRC | Dasatinib | KD | -3.10 | -2.22 (0.09) | 0.91 (0.01) |
| SRC | Dasatinib | (+)-KD-(+) | -3.10 | -2.60 (0.04) | 0.96 (0.02) |
| SRC | Dasatinib | Full SRC | -3.10 | -2.54 (0.09) | 0.97 (0.01) |
| SRC | Ponatinib | KD | -2.40 | -1.76 (0.05) | 0.75 (0.01) |
| SRC | Ponatinib | (+)-KD-(+) | -2.40 | -1.46 (0.08) | 0.72 (0.02) |
| SRC | Ponatinib | Full SRC | -2.40 | -1.59 (0.15) | 0.77 (0.05) |
| SRC | Gefitinib | KD | 0.79 | 0.78 (0.05) | 0.17 (0.01) |
| SRC | Gefitinib | (+)-KD-(+) | 0.79 | 0.98 (0.05) | 0.18 (0.02) |
| SRC | Gefitinib | Full SRC | 0.79 | 0.78 (0.10) | 0.21 (0.03) |
| SRC | Lapatinib | KD | 0.87 | 0.47 (0.07) | 0.09 (0.04) |
| SRC | Lapatinib | (+)-KD-(+) | 0.87 | 0.86 (0.12) | 0.10 (0.01) |
| SRC | Lapatinib | Full SRC | 0.87 | 0.83 (0.11) | 0.12 (0.03) |
| SRC | Osimertinib | KD | 0.91 | 0.69 (0.15) | 0.20 (0.03) |
| SRC | Osimertinib | (+)-KD-(+) | 0.91 | 0.77 (0.16) | 0.20 (0.02) |
| SRC | Osimertinib | Full SRC | 0.91 | 0.60 (0.19) | 0.22 (0.04) |
| SRC | Staurosporine | KD | -2.92 | -1.31 (0.06) | 0.44 (0.01) |
| SRC | Staurosporine | (+)-KD-(+) | -2.92 | -1.04 (0.08) | 0.38 (0.03) |
| SRC | Staurosporine | Full SRC | -2.92 | -1.20 (0.13) | 0.37 (0.06) |
| SRC | Sorafenib | KD | -0.26 | -0.07 (0.11) | 0.46 (0.07) |
| SRC | Sorafenib | (+)-KD-(+) | -0.26 | -0.20 (0.09) | 0.47 (0.02) |
| SRC | Sorafenib | Full SRC | -0.26 | -0.56 (0.13) | 0.61 (0.08) |
| EGFR | Imatinib | KD | 1.08 | 0.05 (0.09) | 0.28 (0.04) |
| EGFR | Imatinib | (+)-KD | 1.08 | 0.33 (0.11) | 0.23 (0.03) |
| EGFR | Imatinib | KD-(+) | 1.08 | -0.07 (0.17) | 0.27 (0.06) |
| EGFR | Imatinib | (+)-KD-(+) | 1.08 | 0.22 (0.18) | 0.20 (0.07) |
| EGFR | Imatinib | Full EGFR | 1.08 | 0.02 (0.22) | 0.26 (0.10) |
| EGFR | Imatinib | ((+)-KD)x2 (Dimer) | 1.08 | 0.36 (0.10) | 0.24 (0.01) |
| EGFR | Dasatinib | KD | 0.26 | -1.14 (0.07) | 0.78 (0.01) |
| EGFR | Dasatinib | (+)-KD | 0.26 | -1.42 (0.11) | 0.82 (0.02) |
| EGFR | Dasatinib | KD-(+) | 0.26 | -1.37 (0.28) | 0.89 (0.01) |
| EGFR | Dasatinib | (+)-KD-(+) | 0.26 | -1.42 (0.14) | 0.88 (0.01) |
| EGFR | Dasatinib | Full EGFR | 0.26 | -1.41 (0.22) | 0.89 (0.01) |
| EGFR | Dasatinib | ((+)-KD)x2 (Dimer) | 0.26 | -1.37 (0.11) | 0.80 (0.02) |
| EGFR | Ponatinib | KD | -0.07 | -1.27 (0.09) | 0.71 (0.01) |
| EGFR | Ponatinib | (+)-KD | -0.07 | -1.41 (0.07) | 0.73 (0.03) |
| EGFR | Ponatinib | KD-(+) | -0.07 | -1.55 (0.15) | 0.81 (0.02) |
| EGFR | Ponatinib | (+)-KD-(+) | -0.07 | -1.57 (0.16) | 0.79 (0.02) |
| EGFR | Ponatinib | Full EGFR | -0.07 | -1.26 (0.19) | 0.77 (0.06) |
| EGFR | Ponatinib | ((+)-KD)x2 (Dimer) | -0.07 | -1.45 (0.11) | 0.73 (0.03) |
| EGFR | Gefitinib | KD | -1.48 | -0.30 (0.10) | 0.46 (0.02) |
| EGFR | Gefitinib | (+)-KD | -1.48 | -0.62 (0.11) | 0.50 (0.03) |
| EGFR | Gefitinib | KD-(+) | -1.48 | -0.67 (0.12) | 0.81 (0.02) |
| EGFR | Gefitinib | (+)-KD-(+) | -1.48 | -0.77 (0.10) | 0.82 (0.02) |
| EGFR | Gefitinib | Full EGFR | -1.48 | -0.88 (0.14) | 0.82 (0.02) |
| EGFR | Gefitinib | ((+)-KD)x2 (Dimer) | -1.48 | -0.61 (0.08) | 0.50 (0.05) |
| EGFR | Lapatinib | KD | -0.96 | -1.16 (0.24) | 0.30 (0.04) |
| EGFR | Lapatinib | (+)-KD | -0.96 | -1.20 (0.10) | 0.36 (0.04) |
| EGFR | Lapatinib | KD-(+) | -0.96 | -1.53 (0.23) | 0.69 (0.05) |
| EGFR | Lapatinib | (+)-KD-(+) | -0.96 | -1.11 (0.18) | 0.59 (0.09) |
| EGFR | Lapatinib | Full EGFR | -0.96 | -1.10 (0.20) | 0.62 (0.10) |
| EGFR | Lapatinib | ((+)-KD)x2 (Dimer) | -0.96 | -1.17 (0.07) | 0.38 (0.03) |
| EGFR | Osimertinib | KD | -1.35 | -1.46 (0.07) | 0.75 (0.01) |
| EGFR | Osimertinib | (+)-KD | -1.35 | -1.27 (0.05) | 0.76 (0.01) |
| EGFR | Osimertinib | KD-(+) | -1.35 | -1.49 (0.16) | 0.86 (0.02) |
| EGFR | Osimertinib | (+)-KD-(+) | -1.35 | -1.37 (0.12) | 0.86 (0.02) |
| EGFR | Osimertinib | Full EGFR | -1.35 | -1.34 (0.25) | 0.83 (0.02) |
| EGFR | Osimertinib | ((+)-KD)x2 (Dimer) | -1.35 | -1.26 (0.10) | 0.75 (0.01) |
| EGFR | Staurosporine | KD | -1.92 | -1.36 (0.05) | 0.47 (0.02) |
| EGFR | Staurosporine | (+)-KD | -1.92 | -1.37 (0.06) | 0.52 (0.01) |
| EGFR | Staurosporine | KD-(+) | -1.92 | -1.50 (0.10) | 0.66 (0.04) |
| EGFR | Staurosporine | (+)-KD-(+) | -1.92 | -1.04 (0.09) | 0.59 (0.03) |
| EGFR | Staurosporine | Full EGFR | -1.92 | -1.04 (0.18) | 0.59 (0.05) |
| EGFR | Staurosporine | ((+)-KD)2x (Dimer) | -1.92 | -1.36 (0.06) | 0.52 (0.01) |
| EGFR | Sorafenib | KD | 0.51 | 0.17 (0.44) | 0.46 (0.07) |
| EGFR | Sorafenib | (+)-KD | 0.51 | -0.10 (0.43) | 0.46 (0.08) |
| EGFR | Sorafenib | KD-(+) | 0.51 | -0.10 (0.53) | 0.54 (0.11) |
| EGFR | Sorafenib | (+)-KD-(+) | 0.51 | -0.43 (0.40) | 0.63 (0.07) |
| EGFR | Sorafenib | Full EGFR | 0.51 | -0.54 (0.18) | 0.65 (0.08) |
| EGFR | Sorafenib | ((+)-KD)x2 (Dimer) | 0.51 | -0.09 (0.44) | 0.46 (0.09) |
| HER2 | Imatinib | KD | 0.98 | 0.30 (0.10) | 0.28 (0.05) |
| HER2 | Imatinib | (+)-KD-(+) | 0.98 | 0.08 (0.23) | 0.22 (0.06) |
| HER2 | Imatinib | Full HER2 | 0.98 | -0.05 (0.26) | 0.24 (0.06) |
| HER2 | Dasatinib | KD | -0.02 | -0.86 (0.11) | 0.72 (0.02) |
| HER2 | Dasatinib | (+)-KD-(+) | -0.02 | -1.48 (0.07) | 0.87 (0.01) |
| HER2 | Dasatinib | Full HER2 | -0.02 | -1.20 (0.25) | 0.89 (0.02) |
| HER2 | Ponatinib | KD | -0.38 | -1.00 (0.16) | 0.29 (0.02) |
| HER2 | Ponatinib | (+)-KD-(+) | -0.38 | -1.03 (0.14) | 0.64 (0.05) |
| HER2 | Ponatinib | Full HER2 | -0.38 | -1.38 (0.14) | 0.70 (0.09) |
| HER2 | Gefitinib | KD | -0.42 | -0.19 (0.09) | 0.42 (0.04) |
| HER2 | Gefitinib | (+)-KD-(+) | -0.42 | -0.52 (0.09) | 0.73 (0.03) |
| HER2 | Gefitinib | Full HER2 | -0.42 | -0.60 (0.31) | 0.77 (0.06) |
| HER2 | Lapatinib | KD | -1.75 | -0.84 (0.19) | 0.29 (0.03) |
| HER2 | Lapatinib | (+)-KD-(+) | -1.75 | -1.12 (0.19) | 0.63 (0.04) |
| HER2 | Lapatinib | Full HER2 | -1.75 | -1.30 (0.35) | 0.63 (0.12) |
| HER2 | Osimertinib | KD | -0.28 | -1.29 (0.04) | 0.71 (0.01) |
| HER2 | Osimertinib | (+)-KD-(+) | -0.28 | -1.47 (0.22) | 0.79 (0.06) |
| HER2 | Osimertinib | Full HER2 | -0.28 | -1.38 (0.21) | 0.75 (0.04) |
| HER2 | Staurosporine | KD | -2.10 | -1.48 (0.03) | 0.48 (0.02) |
| HER2 | Staurosporine | (+)-KD-(+) | -2.10 | -1.32 (0.05) | 0.60 (0.01) |
| HER2 | Staurosporine | Full HER2 | -2.10 | -1.20 (0.18) | 0.66 (0.06) |
| RIPK2 | Staurosporine | KD | -1.66 | -1.37 (0.11) | 0.44 (0.03) |
| RIPK2 | Staurosporine | (+)-KD-(+) | -1.66 | -1.04 (0.06) | 0.53 (0.01) |
| RIPK2 | Staurosporine | Full RIPK2 | -1.66 | -1.23 (0.23) | 0.74 (0.08) |
| BRAF | Imatinib | KD | 1.18 | 0.82 (0.06) | 0.53 (0.01) |
| BRAF | Imatinib | (+)-KD-(+) | 1.18 | 0.73 (0.09) | 0.47 (0.04) |
| BRAF | Imatinib | Full BRAF | 1.18 | 0.66 (0.28) | 0.60 (0.07) |
| BRAF | Dasatinib | KD | 0.88 | -0.39 (0.06) | 0.62 (0.02) |
| BRAF | Dasatinib | (+)-KD-(+) | 0.88 | -0.45 (0.26) | 0.61 (0.06) |
| BRAF | Dasatinib | Full BRAF | 0.88 | -0.78 (0.29) | 0.80 (0.02) |
| BRAF | Ponatinib | KD | 0.26 | -0.57 (0.04) | 0.57 (0.01) |
| BRAF | Ponatinib | (+)-KD-(+) | 0.26 | -0.59 (0.09) | 0.55 (0.02) |
| BRAF | Ponatinib | Full BRAF | 0.26 | -0.68 (0.24) | 0.63 (0.05) |
| BRAF | Gefitinib | KD | 1.08 | 0.90 (0.08) | 0.21 (0.02) |
| BRAF | Gefitinib | (+)-KD-(+) | 1.08 | 0.83 (0.10) | 0.23 (0.07) |
| BRAF | Gefitinib | Full BRAF | 1.08 | 0.94 (0.26) | 0.29 (0.13) |
| BRAF | Lapatinib | KD | 1.15 | -0.32 (0.13) | 0.10 (0.01) |
| BRAF | Lapatinib | (+)-KD-(+) | 1.15 | -0.35 (0.13) | 0.10 (0.03) |
| BRAF | Lapatinib | Full BRAF | 1.15 | -0.52 (0.24) | 0.17 (0.04) |
| BRAF | Osimertinib | KD | 1.02 | -0.53 (0.10) | 0.15 (0.02) |
| BRAF | Osimertinib | (+)-KD-(+) | 1.02 | -0.35 (0.24) | 0.13 (0.02) |
| BRAF | Osimertinib | Full BRAF | 1.02 | -0.15 (0.29) | 0.15 (0.11) |
| BRAF | Staurosporine | KD | -1.55 | -0.79 (0.03) | 0.25 (0.01) |
| BRAF | Staurosporine | (+)-KD-(+) | -1.55 | -0.76 (0.14) | 0.28 (0.03) |
| BRAF | Staurosporine | Full BRAF | -1.55 | -0.49 (0.20) | 0.50 (0.09) |
| BRAF | Sorafenib | KD | -1.55 | -0.43 (0.09) | 0.61 (0.03) |
| BRAF | Sorafenib | (+)-KD-(+) | -1.55 | -0.46 (0.08) | 0.61 (0.03) |
| BRAF | Sorafenib | Full BRAF | -1.55 | -0.72 (0.11) | 0.76 (0.07) |
| BRAF | Dabrafenib | KD | -2.50 | -1.90 (0.07) | 0.87 (0.02) |
| BRAF | Dabrafenib | (+)-KD-(+) | -2.50 | -1.93 (0.10) | 0.87 (0.02) |
| BRAF | Dabrafenib | Full BRAF | -2.50 | -2.57 (0.13) | 0.98 (0.02) |

**Table S5. Pearson correlation coefficients between experimental and predicted log_10_(IC_50_) values stratified by kinase and inhibitor.** Correlation coefficients were calculated separately for each inhibitor and each kinase across the three sequence‑input definitions: kinase domain only (KD), kinase domain with flanking regions [(+)-KD-(+)], and full‑length protein. The row labeled “All” summarizes correlations across all kinase-inhibitor pairs included in the benchmark dataset. Note that one pair involving RIPK2 was excluded from the kinase-specific stratification because a correlation coefficient cannot be mathematically defined for a single data point, resulting in N = 56 for the stratified analysis.

|  |  |  |  | **Region** |  |
| --- | --- | --- | --- | --- | --- |
|  |  | N | **KD** | **(+)-KD-(+)** | **Full-length** |
|  | All | 56 | 0.82 | 0.81 | 0.81 |
| Inhibitor | Dasatinib | 7 | 0.84 | 0.91 | 0.89 |
|  | Gefitinib | 7 | 0.90 | 0.94 | 0.93 |
|  | Imatinib | 7 | 0.84 | 0.93 | 0.87 |
|  | Lapatinib | 7 | 0.74 | 0.79 | 0.77 |
|  | Osimertinib | 7 | 0.82 | 0.83 | 0.87 |
|  | Ponatinib | 7 | 0.79 | 0.77 | 0.79 |
|  | Sorafenib | 6 | 0.65 | 0.37 | 0.52 |
|  | Staurosporine | 8 | 0.37 | 0.38 | 0.49 |
| Kinase | ABL1 | 8 | 0.92 | 0.92 | 0.91 |
|  | BRAF | 9 | 0.74 | 0.76 | 0.73 |
|  | EGFR | 8 | 0.57 | 0.47 | 0.53 |
|  | HER2 | 7 | 0.68 | 0.55 | 0.60 |
|  | KIT | 8 | 0.96 | 0.96 | 0.92 |
|  | PDGFRA | 8 | 0.94 | 0.92 | 0.89 |
|  | SRC | 8 | 0.97 | 0.94 | 0.95 |

**Table S6. Leave-one-kinase-out comparison of binder/non-binder classification using Boltz-2 potency and binding-probability outputs.** Binder/non-binder classification performance was evaluated using leave-one-kinase-out cross-validation for each sequence input definition. Models were constructed using predicted log_10_(IC_50_) alone (Pred log_10_(IC_50_)), predicted binding probability alone (BP), or both outputs (Combined). Experimental kinase-inhibitor pairs with log_10_(IC_50_) < 0 were defined as binders, whereas those with log_10_(IC_50_) ≥ 0 were defined as non-binders. ROC AUC, balanced accuracy, sensitivity, and specificity were calculated from predictions pooled across the held-out kinase folds.

| **Sequence input** | **Model** | **ROC AUC** | **Balanced accuracy** | **Sensitivity** | **Specificity** |
| --- | --- | --- | --- | --- | --- |
| KD | Pred log_10_(IC_50_) | 0.941 | 0.818 | 0.853 | 0.783 |
|  | BP | 0.873 | 0.767 | 0.794 | 0.739 |
|  | Combined | 0.939 | 0.891 | 0.912 | 0.870 |
| (+)-KD-(+) | Pred log_10_(IC_50_) | 0.955 | 0.891 | 0.912 | 0.870 |
|  | BP | 0.900 | 0.796 | 0.853 | 0.739 |
|  | Combined | 0.950 | 0.854 | 0.882 | 0.826 |
| Full-length | Pred log_10_(IC_50_) | 0.941 | 0.877 | 0.971 | 0.783 |
|  | BP | 0.898 | 0.862 | 0.941 | 0.783 |
|  | Combined | 0.939 | 0.884 | 0.941 | 0.826 |

**Table S7. Pearson correlation coefficients between experimental log_10_(IC_50_) values and predicted binding probability scores stratified by kinase and inhibitor.** Correlation coefficients were calculated separately for each inhibitor and each kinase across the three sequence‑input definitions: kinase domain only (KD), kinase domain with flanking regions [(+)-KD-(+)], and full‑length protein. The row labeled “All” summarizes correlations across all kinase–inhibitor pairs included in the benchmark dataset. Note that one pair involving RIPK2 was excluded from the kinase-specific stratification because a correlation coefficient cannot be mathematically defined for a single data point, resulting in N = 56 for the stratified analysis.

|  |  |  |  | **Region** |  |
| --- | --- | --- | --- | --- | --- |
|  |  | **N** | **KD** | **(+)-KD-(+)** | **Full-length** |
|  | All | 56 | -0.71 | -0.68 | -0.71 |
| Inhibitor | Dasatinib | 7 | -0.81 | -0.74 | -0.84 |
|  | Gefitinib | 7 | -0.76 | -0.93 | -0.93 |
|  | Imatinib | 7 | -0.67 | -0.77 | -0.75 |
|  | Lapatinib | 7 | -0.94 | -0.98 | -0.96 |
|  | Osimertinib | 7 | -0.94 | -0.94 | -0.89 |
|  | Ponatinib | 7 | -0.66 | -0.70 | -0.75 |
|  | Sorafenib | 6 | -0.68 | -0.38 | -0.65 |
|  | Staurosporine | 8 | -0.40 | -0.23 | 0.14 |
| Kinase | ABL1 | 8 | -0.82 | -0.80 | -0.88 |
|  | BRAF | 9 | -0.55 | -0.60 | -0.64 |
|  | EGFR | 8 | -0.08 | -0.43 | -0.40 |
|  | HER2 | 7 | 0.05 | -0.30 | -0.34 |
|  | KIT | 8 | -0.95 | -0.93 | -0.93 |
|  | PDGFRA | 8 | -0.94 | -0.88 | -0.89 |
|  | SRC | 8 | -0.87 | -0.84 | -0.78 |

**Table S8. Curated experimental IC₅₀ benchmark dataset for mutant kinase-inhibitor pairs.** Representative IC_50_ values were obtained as the median of publicly available biochemical measurements curated from BindingDB and PubChem BioAssay. IC_50_ values are reported in µM, and log_10_(IC_50_) values were calculated using the µM scale. Duplicate entries were removed. The column *n* indicates the number of independent experimental IC_50_ measurements; only kinase-inhibitor pairs with *n* ≥ 3 were included in the benchmark dataset.

| **Kinase** | **Variant** | **Inhibitor** | ***n*** | **Median IC_50_** | **log_10_(IC_50_)** |
| --- | --- | --- | --- | --- | --- |
| ABL1 | Y253F | Imatinib | 4 | 1.80 | 0.26 |
|  |  | Dasatinib | 5 | 0.006 | -2.22 |
|  |  | Ponatinib | 5 | 0.0018 | -2.75 |
|  | T315I | Imatinib | 7 | 8.50 | 0.93 |
|  |  | Dasatinib | 6 | 3.20 | 0.51 |
|  |  | Ponatinib | 10 | 0.0025 | -2.60 |
|  |  | Staurosporine | 3 | 0.015 | -1.82 |
|  |  | Sorafenib | 3 | 0.98 | -0.01 |
| EGFR | L858R | Gefitinib | 7 | 0.009 | -2.05 |
|  |  | Lapatinib | 4 | 0.07 | -1.16 |
|  |  | Osimertinib | 8 | 0.012 | -1.92 |
|  |  | Staurosporine | 3 | 0.01 | -2.00 |
|  | L858R/T790M | Gefitinib | 6 | 4.80 | 0.68 |
|  |  | Lapatinib | 3 | 3.60 | 0.56 |
|  |  | Osimertinib | 10 | 0.0045 | -2.35 |
|  |  | Staurosporine | 3 | 0.018 | -1.75 |
|  | L858R/T790M/C797S | Osimertinib | 5 | 3.80 | 0.58 |
| BRAF | V600E | Staurosporine | 4 | 0.031 | -1.51 |
|  |  | Sorafenib | 4 | 0.04 | -1.40 |
|  |  | Dabrafenib | 8 | 0.0007 | -3.16 |

**Table S9. Complete Boltz-2 prediction outputs for mutant systems.** Predicted log_10_(IC_50_) values and binding probability (BP) are reported for each mutant kinase-inhibitor pair. Experimental IC_50_ values are given in µM and corresponding log_10_(IC_50_) values are reported. All predicted values were obtained using kinase-domain-only inputs and are reported as the median across ten independent Boltz-2 runs.

| **Kinase** | **Variant** | **Inhibitor** | **Exp log_10_(IC_50_)** | **Pred log_10_(IC_50_)** | **BP** |
| --- | --- | --- | --- | --- | --- |
| ABL1 | Y253F | Imatinib | 0.26 | -0.01 | 0.36 |
| ABL1 | Y253F | Dasatinib | -2.22 | -1.72 | 0.87 |
| ABL1 | Y253F | Ponatinib | -2.75 | -1.73 | 0.84 |
| ABL1 | T315I | Imatinib | 0.93 | 0.42 | 0.42 |
| ABL1 | T315I | Dasatinib | 0.51 | 0.05 | 0.49 |
| ABL1 | T315I | Ponatinib | -2.60 | -1.26 | 0.69 |
| EGFR | L858R | Gefitinib | -2.05 | -0.24 | 0.47 |
| EGFR | L858R | Lapatinib | -1.16 | -0.70 | 0.23 |
| EGFR | L858R | Osimertinib | -1.92 | -1.29 | 0.73 |
| EGFR | L858R | Staurosporine | -2.00 | -1.45 | 0.51 |
| EGFR | L858R/T790M | Gefitinib | 0.68 | 0.08 | 0.40 |
| EGFR | L858R/T790M | Lapatinib | 0.56 | -0.43 | 0.22 |
| EGFR | L858R/T790M | Osimertinib | -2.35 | -1.35 | 0.76 |
| EGFR | L858R/T790M | Staurosporine | -1.75 | -2.06 | 0.73 |
| EGFR | L858R/T790M/C797S | Osimertinib | 0.58 | -0.32 | 0.51 |
| BRAF | V600E | Staurosporine | -1.51 | -0.73 | 0.25 |
| BRAF | V600E | Sorafenib | -1.40 | -0.39 | 0.59 |
| BRAF | V600E | Dabrafenib | -3.16 | -2.00 | 0.88 |

**Table S10. Conformational states of the αC-helix and DFG motif in predicted kinase-inhibitor complex structures.** Predicted αC‑helix and DFG conformational states are shown for each kinase-inhibitor complex. Experimental conformational states were assigned based on available experimental structures, and PDB IDs are provided when applicable. “Consistency” indicates agreement between predicted and experimental conformational states. All predicted structures were generated using kinase domain (KD) sequences of wild‑type kinases, with the representative structure selected as the run closest to the median predicted log_10_(IC_50_) value across ten independent Boltz-2 runs.

| **Kinase** | **Inhibitor** | **αC state** | **DFG state** | **Experimental state** | **PDB ID** | **Consistency** |
| --- | --- | --- | --- | --- | --- | --- |
| ABL1 | Imatinib | IN | OUT | αC-IN, DFG-OUT | 2HYY | Match |
|  | Dasatinib | IN | IN | αC-IN, DFG-IN | 2GQG | Match |
|  | Ponatinib | IN | OUT |  |  |  |
|  | Gefitinib | IN | IN |  |  |  |
|  | Lapatinib | OUT | OUT |  |  |  |
|  | Osimertinib | OUT | OUT |  |  |  |
|  | Staurosporine | IN | IN |  |  |  |
|  | Sorafenib | OUT | OUT |  |  |  |
| PDGFRA | Imatinib | IN | OUT | αC-IN, DFG-OUT | 6JOL | Match |
|  | Dasatinib | IN | OUT |  |  |  |
|  | Ponatinib | IN | OUT |  |  |  |
|  | Gefitinib | IN | OUT |  |  |  |
|  | Lapatinib | IN | OUT |  |  |  |
|  | Osimertinib | IN | OUT |  |  |  |
|  | Staurosporine | IN | IN |  |  |  |
|  | Sorafenib | IN | OUT |  |  |  |
| KIT | Imatinib | IN | OUT | αC-IN, DFG-OUT | 8S16 | Match |
|  | Dasatinib | IN | IN |  |  |  |
|  | Ponatinib | IN | OUT | αC-IN, DFG-OUT | 4U0I | Match |
|  | Gefitinib | IN | IN |  |  |  |
|  | Lapatinib | IN | OUT |  |  |  |
|  | Osimertinib | IN | IN |  |  |  |
|  | Staurosporine | IN | IN | αC-IN, DFG-IN | 8S19 | Match |
|  | Sorafenib | IN | OUT |  |  |  |
| SRC | Imatinib | IN | OUT |  |  |  |
|  | Dasatinib | IN | IN |  |  |  |
|  | Ponatinib | IN | OUT | αC-IN, DFG-OUT | 7OTE | Match |
|  | Gefitinib | OUT | OUT |  |  |  |
|  | Lapatinib | OUT | OUT |  |  |  |
|  | Osimertinib | OUT | OUT |  |  |  |
|  | Staurosporine | OUT | OUT |  |  |  |
|  | Sorafenib | IN | OUT |  |  |  |
| EGFR | Imatinib | IN | OUT |  |  |  |
|  | Dasatinib | OUT | IN |  |  |  |
|  | Ponatinib | IN | OUT |  |  |  |
|  | Gefitinib | IN | IN | αC-IN, DFG-IN | 2ITY | Match |
|  | Lapatinib | OUT | OUT | αC-OUT, DFG-OUT | 1XKK | Match |
|  | Osimertinib | IN | IN | αC-IN, DFG-IN | 4ZAU | Match |
|  | Staurosporine | IN | IN |  |  |  |
|  | Sorafenib | IN | OUT |  |  |  |
| HER2 | Imatinib | IN | OUT |  |  |  |
|  | Dasatinib | IN | IN |  |  |  |
|  | Ponatinib | OUT | IN |  |  |  |
|  | Gefitinib | IN | IN |  |  |  |
|  | Lapatinib | OUT | IN |  |  |  |
|  | Osimertinib | IN | IN |  |  |  |
|  | Staurosporine | IN | IN |  |  |  |
| RIPK2 | Staurosporine | IN | IN | αC-IN, DFG-IN | 5NG2 | Match |
| BRAF | Imatinib | IN | OUT |  |  |  |
|  | Dasatinib | OUT | OUT |  |  |  |
|  | Ponatinib | IN | OUT |  |  |  |
|  | Gefitinib | OUT | OUT |  |  |  |
|  | Lapatinib | OUT | OUT |  |  |  |
|  | Osimertinib | OUT | OUT |  |  |  |
|  | Staurosporine | IN | IN |  |  |  |
|  | Sorafenib | IN | OUT | αC-IN, DFG-OUT | 1UWH | Match |
|  | Dabrafenib | OUT | OUT | αC-OUT, DFG-OUT | 5CSW | Match |

**Table S11. Structural validation metrics for predicted kinase-inhibitor complex models against reference experimental structures.** For each kinase-inhibitor complex, predicted structures generated using different sequence‑input definitions were compared with available experimental reference structures. Structural similarity was assessed using RMSD_KD_ (Cα‑RMSD of the kinase domain) and RMSD_Lig_ (heavy‑atom RMSD of the inhibitor after structural alignment of the kinase domain). Experimental log_10_(IC_50_), predicted log_10_(IC_50_), and binding‑probability (BP) are shown for each model.

| **Kinase** | **Inhibitor** | **Region** | **PDB** | **RMSD_KD_** | **RMSD_Lig_** | **Exp log_10_(IC_50_)** | **Pred log_10_(IC_50_)** | **BP** |
| --- | --- | --- | --- | --- | --- | --- | --- | --- |
| ABL1 | Imatinib | KD | 2HYY | 0.21 | 0.28 | -1.00 | -0.02 | 0.37 |
|  |  | (+)-KD-(+) |  | 0.26 | 0.25 |  | -0.47 | 0.43 |
|  |  | Full ABL1 |  | 0.48 | 0.37 |  | -0.71 | 0.59 |
|  |  | Full BCR-ABL1 |  | 0.50 | 0.42 |  | -0.50 | 0.49 |
|  | Dasatinib | KD | 2GQG | 0.35 | 1.06 | -2.82 | -1.75 | 0.88 |
|  |  | (+)-KD-(+) |  | 0.30 | 1.25 |  | -2.21 | 0.94 |
|  |  | Full ABL1 |  | 0.48 | 1.13 |  | -2.23 | 0.95 |
|  |  | Full BCR-ABL1 |  | 0.54 | 1.23 |  | -2.20 | 0.95 |
| PDGFRA | Imatinib | KD | 6JOL | 0.34 | 0.26 | -1.10 | -1.06 | 0.76 |
|  |  | (+)-KD-(+) |  | 0.45 | 0.36 |  | -1.23 | 0.79 |
|  |  | Full |  | 0.35 | 0.35 |  | -0.77 | 0.72 |
| KIT | Imatinib | KD | 8S16 | 0.35 | 1.16 | -1.05 | -0.90 | 0.70 |
|  |  | (+)-KD-(+) |  | 0.31 | 1.17 |  | -0.95 | 0.61 |
|  |  | Full |  | 0.37 | 1.23 |  | -0.32 | 0.63 |
|  | Ponatinib | KD | 4U0I | 0.34 | 0.49 | -2.00 | -2.04 | 0.90 |
|  |  | (+)-KD-(+) |  | 0.35 | 0.42 |  | -2.04 | 0.90 |
|  |  | Full |  | 0.41 | 0.45 |  | -1.94 | 0.89 |
|  | Staurosporine | KD | 8S19 | 0.50 | 0.78 | -2.42 | -2.29 | 0.90 |
|  |  | (+)-KD-(+) |  | 0.46 | 0.66 |  | -2.31 | 0.92 |
|  |  | Full |  | 0.45 | 0.52 |  | -2.01 | 0.83 |
| SRC | Ponatinib | KD | 7OTE | 0.34 | 0.81 | -2.40 | -1.76 | 0.75 |
|  |  | (+)-KD-(+) |  | 0.32 | 1.00 |  | -1.46 | 0.72 |
|  |  | Full |  | 0.37 | 0.96 |  | -1.59 | 0.77 |
| EGFR | Gefitinib | KD | 2ITY | 0.30 | 1.29 | -1.48 | -0.30 | 0.46 |
|  |  | (+)-KD |  | 0.30 | 1.70 |  | -0.62 | 0.50 |
|  |  | KD-(+) |  | 0.29 | 1.90 |  | -0.67 | 0.81 |
|  |  | (+)-KD-(+) |  | 0.25 | 1.17 |  | -0.77 | 0.82 |
|  |  | Full |  | 0.29 | 1.21 |  | -0.88 | 0.82 |
|  |  | ((+)-KD)×2 |  | 0.28 | 1.48 |  | -0.61 | 0.50 |
|  | Lapatinib | KD | 1XKK | 0.24 | 0.91 | -0.96 | -1.16 | 0.30 |
|  |  | (+)-KD |  | 0.41 | 3.29 |  | -1.20 | 0.36 |
|  |  | (KD)-(+) |  | 0.24 | 2.73 |  | -1.53 | 0.69 |
|  |  | (+)-KD-(+) |  | 0.44 | 3.45 |  | -1.11 | 0.59 |
|  |  | Full |  | 0.42 | 2.97 |  | -1.10 | 0.62 |
|  |  | ((+)-KD)×2 |  | 0.47 | 3.24 |  | -1.17 | 0.38 |
|  | Osimertinib | KD | 4ZAU | 0.23 | 2.65 | -1.35 | -1.46 | 0.75 |
|  |  | (+)-KD |  | 0.24 | 1.91 |  | -1.27 | 0.76 |
|  |  | (KD)-(+) |  | 0.21 | 2.71 |  | -1.49 | 0.86 |
|  |  | (+)-KD-(+) |  | 0.28 | 1.82 |  | -1.37 | 0.86 |
|  |  | Full |  | 0.26 | 2.13 |  | -1.34 | 0.83 |
|  |  | ((+)-KD)×2 |  | 0.26 | 1.72 |  | -1.26 | 0.75 |
| RIPK2 | Staurosporine | KD | 5NG2 | 0.30 | 0.45 | -1.66 | -1.37 | 0.44 |
|  |  | (+)-KD-(+) |  | 0.27 | 0.34 |  | -1.04 | 0.53 |
|  |  | Full |  | 0.37 | 0.45 |  | -1.23 | 0.74 |
| BRAF | Sorafenib | KD | 1UWH | 0.39 | 0.75 | -1.55 | -0.43 | 0.61 |
|  |  | (+)-KD-(+) |  | 0.36 | 0.71 |  | -0.46 | 0.61 |
|  |  | Full |  | 0.31 | 0.72 |  | -0.72 | 0.76 |
|  | Dabrafenib | KD | 5CSW | 0.72 | 0.56 | -2.50 | -1.90 | 0.87 |
|  |  | (+)-KD-(+) |  | 0.60 | 0.71 |  | -1.93 | 0.87 |
|  |  | Full |  | 0.64 | 0.75 |  | -2.57 | 0.98 |

**Table S12. Comparison of ligand-pose accuracy between Boltz-2, AlphaFold 3, and Chai-1 for wild-type kinase-inhibitor complexes.** For each kinase-inhibitor complex, representative structures generated by Boltz-2, AlphaFold 3, and Chai-1 were compared with the corresponding experimental co-crystal structure. Ligand-pose accuracy was assessed using RMSD_Lig_, defined as the heavy-atom RMSD of the inhibitor after alignment of the predicted and experimental structures using kinase-domain Cα atoms.

| **Kinase** | **Inhibitor** | **Reference PDB ID** | **Ligand**  **CCD ID** | **Boltz-2 RMSD_Lig_ (Å)** | **AlphaFold 3 RMSD_Lig_ (Å)** | **Chai-1 RMSD_Lig_ (Å)** |
| --- | --- | --- | --- | --- | --- | --- |
| **ABL1** | **Imatinib** | **2HYY** | **STI** | **0.28** | **0.83** | **4.61** |
| **ABL1** | **Dasatinib** | **2GQG** | **1N1** | **1.06** | **1.87** | **2.15** |
| **PDGFRA** | **Imatinib** | **6JOL** | **STI** | **0.26** | **1.12** | **12.90** |
| **KIT** | **Imatinib** | **8S16** | **STI** | **1.16** | **0.81** | **12.77** |
| **KIT** | **Ponatinib** | **4U0I** | **0LI** | **0.49** | **0.56** | **0.40** |
| **KIT** | **Staurosporine** | **8S19** | **STU** | **0.78** | **0.37** | **0.53** |
| **SRC** | **Ponatinib** | **7OTE** | **0LI** | **0.81** | **0.98** | **0.74** |
| **EGFR** | **Gefitinib** | **2ITY** | **IRE** | **1.29** | **1.11** | **1.92** |
| **EGFR** | **Lapatinib** | **1XKK** | **FMM** | **0.91** | **0.92** | **2.99** |
| **EGFR** | **Osimertinib** | **4ZAU** | **YY3** | **2.65** | **1.16** | **1.83** |
| **BRAF** | **Sorafenib** | **1UWH** | **BAX** | **0.75** | **1.07** | **0.74** |
| **BRAF** | **Dabrafenib** | **5CSW** | **P06** | **0.56** | **0.40** | **0.73** |

T**able S13. Time-resolved structural overlap audit of exact wild-type kinase-inhibitor complexes relative to the Boltz-2 structural training cutoff, with ligand-pose accuracy of Boltz-2, AlphaFold 3, and Chai-1.** For each of the 12 benchmark complexes, publicly available experimental structures containing the same wild-type human kinase and the same inhibitor were identified. PDB release dates were used to determine whether exact matching complexes had been publicly released before the stated Boltz-2 structural training cutoff of 1 June 2023. Structures containing mutations in the benchmark kinase or a different protein target were excluded. Eleven of the 12 kinase-inhibitor pairs had at least one exact wild-type complex released before the cutoff, indicating potential direct structural training exposure. KIT-Staurosporine was the only pair for which no pre-cutoff exact wild-type complex was identified. Ligand-pose accuracy was assessed using RMSD_Lig_ for the representative Boltz-2, AlphaFold 3, and Chai-1 structures.

| **No** | **Kinase** | **Inhibitor** | **Reference PDB ID** | **Reference PDB release date** | **Exact WT complex PDB ID(s) released before 1 Jun 2023** | **Release date(s)** | **No. of exact WT complexes released before cutoff** | **Potential direct structural training exposure** | **Boltz-2 RMSD_Lig_ (Å)** | **AlphaFold 3 RMSD_Lig_ (Å)** | **Chai-1 RMSD_Lig_ (Å)** |
| --- | --- | --- | --- | --- | --- | --- | --- | --- | --- | --- | --- |
| 1 | ABL1 | Imatinib | 2HYY | 2007-01-16 | 2HYY, 3PYY, 6NPE, 6NPU, 6NPV, 7N9G | 2007-01-16; 2011-03-09; 2019-03-13; 2019-03-13; 2019-03-13; 2022-04-27 | 6 | Yes | 0.28 | 0.83 | 4.61 |
| 2 | ABL1 | Dasatinib | 2GQG | 2006-11-21 | 2GQG, 4XEY, 7N9G | 2006-11-21; 2015-04-01; 2022-04-27 | 3 | Yes | 1.06 | 1.87 | 2.15 |
| 3 | PDGFRA | Imatinib | 6JOL | 2020-03-25 | 6JOL | 2020-03-25 | 1 | Yes | 0.26 | 1.12 | 12.90 |
| 4 | KIT | Imatinib | 8S16 | 2025-09-03 | 1T46 | 2004-06-15 | 1 | Yes | 1.16 | 0.81 | 12.77 |
| 5 | KIT | Ponatinib | 4U0I | 2014-10-08 | 4U0I | 2014-10-08 | 1 | Yes | 0.49 | 0.56 | 0.40 |
| 6 | KIT | Staurosporine | 8S19 | 2025-09-03 | None | - | 0 | No | 0.78 | 0.37 | 0.53 |
| 7 | SRC | Ponatinib | 7OTE | 2022-06-22 | 7OTE | 2022-06-22 | 1 | Yes | 0.81 | 0.98 | 0.74 |
| 8 | EGFR | Gefitinib | 2ITY | 2007-04-03 | 2ITY, 4WKQ | 2007-04-03; 2014-11-05 | 2 | Yes | 1.29 | 1.11 | 1.92 |
| 9 | EGFR | Lapatinib | 1XKK | 2004-12-07 | 1XKK | 2004-12-07 | 1 | Yes | 0.91 | 0.92 | 2.99 |
| 10 | EGFR | Osimertinib | 4ZAU | 2015-11-11 | 4ZAU, 6JXT | 2015-11-11; 2020-04-29 | 2 | Yes | 2.65 | 1.16 | 1.83 |
| 11 | BRAF | Sorafenib | 1UWH | 2004-03-19 | 1UWH | 2004-03-19 | 1 | Yes | 0.75 | 1.07 | 0.74 |
| 12 | BRAF | Dabrafenib | 5CSW | 2016-03-09 | 5CSW, 6V2U | 2016-03-09; 2020-12-02 | 2 | Yes | 0.56 | 0.4 | 0.73 |

**Table S14. Record-level provenance of affinity measurements for the seven most extensively characterized kinase-inhibitor pairs.** All kinase-inhibitor pairs in the benchmark supported by at least 10 curated IC_50_ measurements were included. Database identifiers, IC_50_ values, dates recorded in BindingDB, BindingDB DOI identifiers, and available ChEMBL assay/source identifiers are shown. A dash indicates that no corresponding assay/source identifier was identified. The representative activity for each kinase–inhibitor pair was calculated as the median of the curated IC_50_ measurements, as described in Methods.

| **Kinase** | **Inhibitor** | **Database record** | **IC_50_ (μM)** | **Date in BindingDB** | **BindingDB DOI** | **Assay/source ID** |
| --- | --- | --- | --- | --- | --- | --- |
| ABL1 | Imatinib | BindingDB BDBM13530 | 0.020 | 4/13/2014 | 10.7270/Q2W37XPH | ChEMBL_970146 (CHEMBL2406231) |
|  | Imatinib | BindingDB BDBM13530 | 0.040 | 11/10/2009 | 10.7270/Q29K49XM | ChEMBL_321182 (CHEMBL882911) |
|  | Imatinib | BindingDB BDBM13530 | 0.043 | 2/23/2021 | 10.7270/Q2M33062 | ChEMBL_1882010 (CHEMBL4383509) |
|  | Imatinib | BindingDB BDBM13530 | 0.098 | 1/11/2010 | 10.7270/Q2DN44TT | ChEMBL_555674 (CHEMBL955828) |
|  | Imatinib | BindingDB BDBM13530 | 0.098 | 9/24/2013 | 10.7270/Q2V40WH7 | ChEMBL_937547 (CHEMBL2320772) |
|  | Imatinib | BindingDB BDBM13530 | 0.110 | 1/11/2010 | 10.7270/Q20001WP | ChEMBL_491675 (CHEMBL946393) |
|  | Imatinib | BindingDB BDBM13530 | 0.170 | 9/4/2020 | 10.7270/Q2542RK1 | ChEMBL_1538989 (CHEMBL3737992) |
|  | Imatinib | BindingDB BDBM13530 | 0.188 | 6/10/2011 | 10.7270/Q2CC10WV | ChEMBL_660979 (CHEMBL1249646) |
|  | Imatinib | BindingDB BDBM13530 | 0.190 | 9/24/2013 | 10.7270/Q24T6KPD | ChEMBL_941939 (CHEMBL2330096) |
|  | Imatinib | BindingDB BDBM13530 | 0.200 | 4/10/2016 | 10.7270/Q2C82C03 | ChEMBL_1464736 (CHEMBL3407087) |
|  | Dasatinib | BindingDB BDBM13216 | 0.000260 | 9/24/2013 | 10.7270/Q2V40WH7 | ChEMBL_937547 (CHEMBL2320772) |
|  | Dasatinib | BindingDB BDBM13216 | 0.000300 | 6/23/2023 | 10.7270/Q25M69R7 | ChEMBL_2228892 (CHEMBL5142405) |
|  | Dasatinib | PubChem AID 2193600 | 0.000600 | 10/13/2025 | 10.7270/Q2D79H45 | ChEMBL_2578573 |
|  | Dasatinib | PubChem AID 2168182 | 0.000700 | 10/13/2025 | 10.7270/Q2D79H45 | ChEMBL_2578586 |
|  | Dasatinib | BindingDB BDBM13216 | 0.001000 | 3/28/2022 | 10.7270/Q21J9FHZ | ChEMBL_2074185 (CHEMBL4729719) |
|  | Dasatinib | PubChem AID 2168181 | 0.001100 | 10/13/2025 | 10.7270/Q2N302PB | ChEMBL_2543972 |
| ABL1 | Dasatinib | BindingDB BDBM13216 | 0.001500 | 12/20/2023 | 10.7270/Q2PV6QJK | — |
|  | Dasatinib | BindingDB BDBM13216 | 0.003100 | 2/26/2021 | 10.7270/Q29P353V | ChEMBL_1909785 (CHEMBL4412231) |
|  | Dasatinib | BindingDB BDBM13216 | 0.004000 | 8/21/2018 | 10.7270/Q2ZP484T | ChEMBL_1628686 (CHEMBL3871312) |
|  | Dasatinib | BindingDB BDBM13216 | 0.004200 | 11/11/2009 | 10.7270/Q25D8RK2 | ChEMBL_451331 (CHEMBL901542) |
|  | Dasatinib | BindingDB BDBM13216 | 0.014000 | 12/15/2012 | 10.7270/Q24J0G05 | ChEMBL_477211 (CHEMBL924604) |
|  | Dasatinib | BindingDB BDBM13216 | 0.138000 | 6/22/2023 | 10.7270/Q20Z779V | ChEMBL_2218622 (CHEMBL5131956) |
|  | Ponatinib | BindingDB BDBM50322535 | 0.00031 | 8/11/2021 | 10.7270/Q2377D68 | ChEMBL_1972825 (CHEMBL4605643) |
|  | Ponatinib | BindingDB BDBM50322535 | 0.00033 | 9/24/2013 | 10.7270/Q2V40WH7 | ChEMBL_937547 (CHEMBL2320772) |
|  | Ponatinib | BindingDB BDBM50322535 | 0.00037 | 6/19/2023 | 10.7270/Q2Z89HCM | ChEMBL_2196453 (CHEMBL5108969) |
|  | Ponatinib | BindingDB BDBM50322535 | 0.00037 | 3/21/2022 | 10.7270/Q2GM8BXF | ChEMBL_2014794 (CHEMBL4668372) |
|  | Ponatinib | BindingDB BDBM50322535 | 0.00037 | 3/27/2022 | 10.7270/Q2WM1J37 | ChEMBL_2066532 (CHEMBL4721785) |
|  | Ponatinib | BindingDB BDBM50322535 | 0.00037 | 6/22/2023 | 10.7270/Q2MK6HXN | ChEMBL_2220460 (CHEMBL5133794) |
|  | Ponatinib | BindingDB BDBM50322535 | 0.00037 | 11/21/2010 | 10.7270/Q27P8ZKX | ChEMBL_641699 (CHEMBL1176866) |
|  | Ponatinib | BindingDB BDBM50322535 | 0.00037 | 12/20/2024 | 10.7270/Q2C82FRS | ChEMBL_2362738 |
|  | Ponatinib | BindingDB BDBM50322535 | 0.00037 | 5/24/2019 | 10.7270/Q2445PQT | ChEMBL_1649214 (CHEMBL3998348) |
|  | Ponatinib | PubChem AID 1886528 / BindingDB BDBM50322535 | 0.00220 | 6/23/2023 | 10.7270/Q25M69R7 | ChEMBL_2228890 (CHEMBL5142403) |
|  | Ponatinib | PubChem AID 759168 / BindingDB BDBM50322535 | 0.00290 | 4/13/2014 | 10.7270/Q2W37XPH | ChEMBL_970146 (CHEMBL2406231) |
|  | Ponatinib | BindingDB BDBM50322535 | 0.00860 | 11/21/2010 | 10.7270/Q27P8ZKX | ChEMBL_641693 (CHEMBL1176860) |
|  | Ponatinib | BindingDB BDBM50322535 | 0.00860 | 12/5/2011 | 10.7270/Q21G0MM8 | ChEMBL_751378 (CHEMBL1785339) |
| SRC | Dasatinib | BindingDB BDBM13216 | 0.000210 | 6/19/2023 | 10.7270/Q2Z89HCM | ChEMBL_2196448 (CHEMBL5108964) |
|  | Dasatinib | BindingDB BDBM13216 | 0.000300 | 5/6/2016 | 10.7270/Q2J38V91 | ChEMBL_1476339 (CHEMBL3429862) |
| SRC | Dasatinib | BindingDB BDBM13216 | 0.000500 | 10/2/2017 | 10.7270/Q2B85B2D | ChEMBL_1587941 (CHEMBL3824531) |
|  | Dasatinib | BindingDB BDBM13216 | 0.000600 | 11/18/2010 | 10.7270/Q2VD6ZK0 | ChEMBL_614491 (CHEMBL1110559) |
|  | Dasatinib | BindingDB BDBM13216 | 0.000800 | 4/18/2024 | 10.7270/Q2FB5754 | ChEMBL_2267361 |
|  | Dasatinib | BindingDB BDBM13216 | 0.000900 | 2/26/2021 | 10.7270/Q29P353V | ChEMBL_1909792 (CHEMBL4412238) |
|  | Dasatinib | BindingDB BDBM13216 | 0.001000 | 7/20/2012 | 10.7270/Q2NG4R1D | ChEMBL_776799 (CHEMBL1913896) |
|  | Dasatinib | BindingDB BDBM13216 | 0.001500 | 11/11/2009 | 10.7270/Q25D8RK2 | ChEMBL_451332 (CHEMBL901543) |
|  | Dasatinib | PubChem AID 2193652 | 0.006000 | 10/13/2025 | 10.7270/Q2D79H45 | ChEMBL_2578625 |
|  | Dasatinib | BindingDB BDBM13216 | 0.014000 | 1/11/2010 | 10.7270/Q2V9880T | ChEMBL_563100 (CHEMBL1022370) |
| EGFR | Gefitinib | BindingDB BDBM5447 | 0.000320 | 8/23/2022 | 10.7270/Q2SQ946R | ChEMBL_2121823 (CHEMBL4830970) |
|  | Gefitinib | BindingDB BDBM5447 | 0.001000 | 8/2/2019 | 10.7270/Q2FB556R | ChEMBL_1659894 (CHEMBL4009506) |
|  | Gefitinib | BindingDB BDBM5447 | 0.005400 | 12/11/2012 | 10.7270/Q2GF0VB8 | ChEMBL_443788 (CHEMBL892951) |
|  | Gefitinib | BindingDB BDBM5447 | 0.012000 | 7/6/2017 | 10.7270/Q20G3N1W | ChEMBL_1560433 (CHEMBL3776094) |
|  | Gefitinib | BindingDB BDBM5447 | 0.023000 | 9/1/2020 | 10.7270/Q2SF3045 | ChEMBL_983491 (CHEMBL2429426) |
|  | Gefitinib | BindingDB BDBM5447 | 0.029000 | 2/26/2021 | 10.7270/Q21V5JFW | ChEMBL_1913522 (CHEMBL4416105) |
|  | Gefitinib | BindingDB BDBM5447 | 0.037000 | 3/28/2022 | 10.7270/Q21J9FHZ | ChEMBL_2074183 (CHEMBL4729717) |
|  | Gefitinib | BindingDB BDBM5447 | 0.045000 | 4/19/2024 | 10.7270/Q2F193Z6 | ChEMBL_2270906 |
|  | Gefitinib | BindingDB BDBM5447 | 0.060000 | 2/23/2021 | 10.7270/Q2H41VT4 | ChEMBL_1882927 (CHEMBL4384426) |
|  | Gefitinib | BindingDB BDBM5447 | 0.076000 | 3/13/2016 | 10.7270/Q26W9CPC | ChEMBL_1433615 (CHEMBL3385194) |
|  | Gefitinib | BindingDB BDBM5447 | 0.127000 | 9/20/2017 | 10.7270/Q28S4RVV | ChEMBL_1583975 (CHEMBL3815441) |
|  | Gefitinib | BindingDB BDBM5447 | 0.190000 | 12/29/2019 | 10.7270/Q28W3GS9 | ChEMBL_1690842 |
| EGFR | Osimertinib | PubChem AID 1956454 / BindingDB Reactant set 51528519 | 0.000240 | 4/22/2024 | 10.7270/Q2X92GK8 | ChEMBL_2299669 |
|  | Osimertinib | PubChem AID 1771676 / BindingDB Reactant set 51421146 | 0.000620 | 8/23/2022 | 10.7270/Q2SQ946R | ChEMBL_2121824 (CHEMBL4830971) |
|  | Osimertinib | PubChem AID 1391281 / BindingDB Reactant set 51124592 | 0.001400 | 8/17/2020 | 10.7270/Q2H41V28 | ChEMBL_1770732 (CHEMBL4222844) |
|  | Osimertinib | PubChem AID 1441910 / BindingDB Reactant set 50734814 | 0.001600 | 8/2/2019 | 10.7270/Q2FB556R | ChEMBL_1659894 (CHEMBL4009506) |
|  | Osimertinib | PubChem AID 1898665 / BindingDB Reactant set 51495708 | 0.017000 | 6/24/2023 | 10.7270/Q2Q52TQZ | ChEMBL_2241031 (CHEMBL5155241) |
|  | Osimertinib | PubChem AID 2232927 | 0.070000 | 6/5/2026 | 10.7270/Q2ZK5NJ3 | ChEMBL_2627060 |
|  | Osimertinib | PubChem AID 1583836 / BindingDB Reactant set 51275646 | 0.114000 | 2/23/2021 | 10.7270/Q2WH2TCG | ChEMBL_1880799 (CHEMBL4382298) |
|  | Osimertinib | PubChem AID 1909243 / BindingDB Reactant set 51502549 | 0.120600 | 6/25/2023 | 10.7270/Q2S75MFB | ChEMBL_2251609 (CHEMBL5165819) |
|  | Osimertinib | PubChem AID 1730451 / BindingDB Reactant set 51392746 | 0.152300 | 3/29/2022 | 10.7270/Q2X06BR8 | ChEMBL_2080588 (CHEMBL4736379) |
|  | Osimertinib | BindingDB record | 0.158000 | 6/5/2026 | 10.7270/Q2445SB4 | ChEMBL_2628608 |
| HER2 | Lapatinib | BindingDB BDBM5445 | 0.003000 | 11/17/2010 | 10.7270/Q25B02K2 | ChEMBL_622912 (CHEMBL1116651) |
|  | Lapatinib | BindingDB BDBM5445 | 0.007600 | 6/23/2019 | 10.7270/Q21C200G | — |
|  | Lapatinib | PubChem AID 2125440 | 0.010200 | 11/18/2010 | 10.7270/Q2VD6ZK0 | ChEMBL_614502 (CHEMBL1111475) |
| HER2 | Lapatinib | PubChem AID 2144755 | 0.013000 | 8/14/2021 | 10.7270/Q2G44TV0 | ChEMBL_1994719 (CHEMBL4628614) |
|  | Lapatinib | BindingDB BDBM5445 | 0.015000 | 6/5/2026 | 10.7270/Q20P14W6 | ChEMBL_2638316 |
|  | Lapatinib | BindingDB BDBM5445 | 0.023100 | 6/21/2021 | 10.7270/Q2QJ7MCP | — |
|  | Lapatinib | BindingDB BDBM5445 | 0.033000 | 2/19/2021 | 10.7270/Q2NG4TZB | ChEMBL_1829743 (CHEMBL4329617) |
|  | Lapatinib | BindingDB BDBM5445 | 0.060000 | 7/22/2012 | 10.7270/Q2J966SR | ChEMBL_791586 (CHEMBL1930723) |
|  | Lapatinib | BindingDB BDBM5445 | 0.124000 | 11/17/2010 | 10.7270/Q25B02K2 | ChEMBL_622911 (CHEMBL1116650) |
|  | Lapatinib | BindingDB BDBM5445 | 0.540000 | 5/24/2017 | 10.7270/Q2K35WH9 | ChEMBL_1543610 (CHEMBL3750871) |

**Table S15. Comparison of experimental potency, Boltz-2 predictions, and established affinity-scoring baselines across 57 wild-type kinase–inhibitor pairs.** Experimental log_10_(IC_50_) values are shown together with Boltz-2 predicted log_10_(IC_50_), RTMScore, MM/GBSA, and MM/PBSA results. MM/GBSA and MM/PBSA values are reported in kcal/mol as mean ± SD over snapshots sampled from the 3.1-5.0 ns portion of each 5.0-ns molecular-dynamics trajectory at 0.1-ns intervals (20 snapshots). Ligand RMSD was calculated using the same snapshots after structural alignment of the kinase domain to the initial Boltz-2-predicted complex; ligand heavy atoms were not fitted separately, and the initial Boltz-2 ligand pose was used as the reference. Ligand RMSD values therefore quantify positional stability relative to the initial predicted pose rather than pose accuracy against an experimental structure. RTMScore is reported as the raw score (higher values indicate stronger predicted binding), whereas more negative MM/GBSA and MM/PBSA energies indicate stronger predicted binding.

| **Kinase** | **Inhibitor** | **Experimental log_10_(IC_50_)** | **Boltz-2 predicted log_10_(IC_50_)** | **Boltz-2 SD** | **RTMScore** | **MMGBSA (kcal/mol)** | **MMGBSA SD** | **MMPBSA (kcal/mol)** | **MMPBSA SD** | **Ligand RMSD mean (Å)** | **Ligand RMSD SD (Å)** |
| --- | --- | --- | --- | --- | --- | --- | --- | --- | --- | --- | --- |
| ABL1 | Imat | -1.00 | -0.02 | 0.05 | 163.99 | -64.668 | 3.401 | -19.765 | 2.841 | 1.294 | 0.236 |
|  | Dasa | -2.82 | -1.75 | 0.07 | 181.25 | -51.247 | 3.698 | -16.208 | 2.871 | 1.318 | 0.228 |
|  | Pona | -3.30 | -1.76 | 0.05 | 242.30 | -63.728 | 3.669 | -19.106 | 3.386 | 1.323 | 0.287 |
|  | Gefi | 0.93 | 1.02 | 0.09 | 136.75 | -32.445 | 5.258 | -9.299 | 3.149 | 3.044 | 0.505 |
|  | Osim | 0.89 | 0.25 | 0.09 | 96.230 | -31.242 | 4.177 | -3.944 | 3.366 | 1.903 | 0.369 |
|  | Lapa | 0.96 | 0.10 | 0.14 | 137.440 | -60.448 | 2.887 | -18.442 | 3.372 | 1.691 | 0.195 |
|  | Stau | -2.22 | -0.73 | 0.39 | 157.670 | -53.612 | 3.730 | -16.538 | 3.096 | 0.731 | 0.168 |
|  | Sora | -0.37 | -0.45 | 0.18 | 161.676 | -53.315 | 2.193 | -14.252 | 2.724 | 0.658 | 0.135 |
| EGFR | Imat | 1.08 | 0.05 | 0.09 | 120.370 | -66.579 | 3.693 | -22.540 | 2.422 | 1.617 | 0.166 |
|  | Dasa | 0.26 | -1.14 | 0.07 | 134.023 | -43.737 | 3.008 | -12.322 | 4.020 | 1.966 | 0.503 |
|  | Pona | -0.07 | -1.27 | 0.09 | 181.239 | -68.590 | 4.129 | -20.272 | 2.322 | 1.939 | 0.308 |
|  | Gefi | -1.48 | -0.30 | 0.10 | 146.837 | -42.474 | 3.066 | -14.965 | 2.709 | 1.773 | 0.173 |
| EGFR | Osim | -1.35 | -1.46 | 0.07 | 127.631 | -39.351 | 4.767 | -9.185 | 3.646 | 3.839 | 0.709 |
|  | Lapa | -0.96 | -1.16 | 0.24 | 157.453 | -54.993 | 4.397 | -21.328 | 3.208 | 1.868 | 0.469 |
|  | Stau | -1.92 | -1.36 | 0.05 | 158.540 | -51.860 | 2.913 | -15.369 | 3.550 | 0.749 | 0.185 |
|  | Sora | 0.51 | 0.17 | 0.44 | 126.415 | -54.737 | 3.455 | -17.421 | 2.663 | 1.506 | 0.233 |
| BRAF | Imat | 1.18 | 0.82 | 0.06 | 123.996 | -72.472 | 6.745 | -21.334 | 3.559 | 1.465 | 0.179 |
|  | Dasa | 0.88 | -0.39 | 0.06 | 111.027 | -40.850 | 3.119 | -6.752 | 2.552 | 3.410 | 0.596 |
|  | Pona | 0.26 | -0.57 | 0.04 | 169.404 | -61.543 | 4.276 | -17.231 | 4.292 | 1.161 | 0.177 |
|  | Gefi | 1.08 | 0.90 | 0.08 | 100.892 | -38.243 | 3.615 | -12.967 | 3.257 | 1.565 | 0.252 |
|  | Osim | 1.02 | -0.53 | 0.10 | 77.714 | -38.213 | 2.881 | -6.047 | 3.368 | 2.305 | 0.356 |
|  | Lapa | 1.15 | -0.32 | 0.13 | 120.553 | -49.794 | 4.269 | -14.801 | 3.186 | 1.964 | 0.289 |
|  | Stau | -1.55 | -0.79 | 0.03 | 104.460 | -41.960 | 3.196 | -12.428 | 3.171 | 1.461 | 0.567 |
|  | Sora | -1.55 | -0.43 | 0.09 | 143.792 | -58.761 | 3.608 | -20.747 | 4.075 | 0.672 | 0.167 |
|  | Dabr | -2.50 | -1.90 | 0.07 | 127.566 | -63.664 | 4.445 | -18.600 | 3.707 | 0.946 | 0.197 |
| KIT | Imat | -1.05 | -0.90 | 0.33 | 129.288 | -70.586 | 3.898 | -19.997 | 3.697 | 1.694 | 0.218 |
|  | Dasa | -1.75 | -2.13 | 0.23 | 157.434 | -54.546 | 5.333 | -21.674 | 4.401 | 1.311 | 0.418 |
|  | Pona | -2.00 | -2.04 | 0.10 | 223.433 | -67.0438 | 3.833 | -19.079 | 3.832 | 1.131 | 0.252 |
|  | Gefi | 0.58 | 0.61 | 0.08 | 155.179 | -41.531 | 3.358 | -12.416 | 2.889 | 1.971 | 0.377 |
|  | Osim | 0.73 | 0.64 | 0.47 | 134.684 | -46.342 | 3.098 | -9.320 | 2.925 | 1.867 | 0.344 |
|  | Lapa | 0.74 | -0.20 | 0.30 | 122.847 | -49.239 | 4.055 | -13.107 | 3.666 | 2.846 | 0.338 |
|  | Stau | -2.42 | -2.29 | 0.10 | 166.463 | -49.389 | 4.039 | -14.313 | 3.676 | 0.916 | 0.210 |
| KIT | Sora | -1.16 | -1.07 | 0.15 | 179.942 | -58.902 | 3.701 | -21.865 | 3.677 | 0.962 | 0.187 |
| PDGFRA | Imat | -1.10 | -1.06 | 0.12 | 136.368 | -62.242 | 2.931 | -16.586 | 2.238 | 0.890 | 0.181 |
|  | Dasa | -1.35 | -2.28 | 0.12 | 152.168 | -51.446 | 4.211 | -17.729 | 2.913 | 1.470 | 0.246 |
|  | Pona | -1.92 | -2.27 | 0.10 | 220.713 | -80.208 | 3.785 | -26.444 | 3.667 | 1.818 | 0.218 |
|  | Gefi | 0.72 | 0.26 | 0.17 | 141.409 | -40.523 | 3.920 | -12.344 | 3.106 | 3.765 | 0.765 |
|  | Osim | 0.79 | 0.25 | 0.35 | 119.443 | -42.198 | 4.261 | -9.263 | 4.517 | 1.580 | 0.210 |
|  | Lapa | 0.83 | -0.55 | 0.19 | 157.891 | -53.407 | 2.970 | -14.273 | 3.665 | 2.753 | 0.577 |
|  | Stau | -2.35 | -2.58 | 0.09 | 171.532 | -50.529 | 2.016 | -15.042 | 1.585 | 0.616 | 0.219 |
|  | Sora | -1.00 | -1.55 | 0.11 | 169.572 | -60.666 | 2.570 | -20.516 | 2.477 | 0.621 | 0.161 |
| SRC | Imat | 0.86 | 0.40 | 0.05 | 159.456 | -72.301 | 4.067 | -20.057 | 3.028 | 1.706 | 0.175 |
|  | Dasa | -3.10 | -2.22 | 0.09 | 176.798 | -49.733 | 4.604 | -19.260 | 3.285 | 1.318 | 0.224 |
|  | Pona | -2.40 | -1.76 | 0.05 | 245.315 | -72.960 | 3.888 | -19.796 | 3.485 | 0.970 | 0.150 |
|  | Gefi | 0.79 | 0.78 | 0.05 | 122.903 | -35.175 | 2.648 | -9.904 | 3.056 | 2.423 | 0.468 |
|  | Osim | 0.91 | 0.69 | 0.15 | 110.841 | -32.056 | 3.498 | -6.993 | 2.682 | 3.515 | 0.728 |
|  | Lapa | 0.87 | 0.47 | 0.07 | 134.428 | -46.396 | 4.244 | -12.888 | 4.297 | 2.911 | 0.513 |
|  | Stau | -2.92 | -1.31 | 0.06 | 154.508 | -46.338 | 3.959 | -12.935 | 3.746 | 1.367 | 0.453 |
|  | Sora | -0.26 | -0.07 | 0.11 | 159.739 | -58.669 | 3.816 | -21.242 | 3.217 | 0.838 | 0.133 |
| HER2 | Imat | 0.98 | 0.30 | 0.10 | 111.368 | -67.537 | 5.381 | -21.298 | 3.982 | 1.953 | 0.268 |
|  | Dasa | -0.02 | -0.86 | 0.11 | 133.624 | -47.405 | 2.846 | -13.243 | 2.506 | 1.607 | 0.263 |
|  | Pona | -0.38 | -1.00 | 0.16 | 160.154 | -40.220 | 3.102 | -11.382 | 3.005 | 1.467 | 0.214 |
| HER2 | Gefi | -0.42 | -0.19 | 0.09 | 149.154 | -43.544 | 3.469 | -11.160 | 2.884 | 2.496 | 0.332 |
|  | Osim | -0.28 | -1.29 | 0.04 | 122.541 | -42.480 | 4.499 | -10.151 | 2.438 | 2.472 | 0.395 |
|  | Lapa | -1.75 | -0.84 | 0.19 | 120.480 | -51.823 | 3.359 | -22.381 | 2.650 | 4.098 | 0.695 |
|  | Stau | -2.10 | -1.48 | 0.03 | 164.451 | -49.696 | 3.140 | -16.200 | 3.296 | 1.304 | 0.239 |
|  | Stau | -1.66 | -1.23 | 0.23 | 141.914 | -55.400 | 3.934 | -15.993 | 4.285 | 1.218 | 0.173 |

**Table S16. Global correlations between predicted affinity scores and experimental log_10_(IC_50_) values.** Pearson and Spearman correlation coefficients were calculated across all 57 wild-type kinase-inhibitor pairs. For RTMScore, higher scores indicate stronger predicted binding, whereas lower experimental log_10_(IC_50_) values indicate stronger activity; therefore, favorable agreement is reflected by negative correlation coefficients.

| **Method** | **n** | **Pearson *r*** | **Pearson *p*** | **Spearman *ρ*** | **Spearman *p*** |
| --- | --- | --- | --- | --- | --- |
| **Boltz-2** | 57 | 0.820 | 5.94×10^-15^ | 0.816 | 1.02×10^-14^ |
| **RTMScore** | 57 | -0.611 | 4.47×10^-7^ | -0.624 | 2.14×10^-7^ |
| **MMPBSA** | 57 | 0.385 | 0.00313 | 0.304 | 0.0215 |
| **MMGBSA** | 57 | 0.268 | 0.0441 | 0.250 | 0.0605 |

**Table S17. Kinase-wise correlations between affinity scores and experimental log_10_(IC_50_) values.** Pearson and Spearman correlations were calculated separately for each kinase using the wild-type inhibitor pairs. RIPK2 was excluded because only one wild-type inhibitor pair was available. For RTMScore, higher scores indicate stronger predicted binding, whereas lower experimental log_10_(IC_50_) values indicate stronger activity; therefore, favorable agreement is reflected by negative correlation coefficients.

| **Kinase** | **Method** | **n** | **Pearson r** | **Pearson p** | **Spearman ρ** | **Spearman p** |
| --- | --- | --- | --- | --- | --- | --- |
| ABL1 | Boltz-2 | 8 | 0.916 | 0.0014 | 0.905 | 0.0020 |
|  | RTMScore | 8 | -0.841 | 0.0089 | -0.833 | 0.0102 |
|  | MM/PBSA | 8 | 0.578 | 0.1332 | 0.405 | 0.3199 |
|  | MM/GBSA | 8 | 0.542 | 0.1652 | 0.333 | 0.4198 |
| BRAF | Boltz-2 | 9 | 0.738 | 0.0233 | 0.845 | 0.0041 |
|  | RTMScore | 9 | -0.279 | 0.4678 | -0.343 | 0.3660 |
|  | MM/PBSA | 9 | 0.382 | 0.3100 | 0.025 | 0.9489 |
|  | MM/GBSA | 9 | 0.262 | 0.4962 | 0.134 | 0.7313 |
| EGFR | Boltz-2 | 8 | 0.572 | 0.1386 | 0.667 | 0.0710 |
|  | RTMScore | 8 | -0.396 | 0.3309 | -0.619 | 0.1017 |
|  | MM/PBSA | 8 | -0.458 | 0.2536 | -0.476 | 0.2329 |
|  | MM/GBSA | 8 | -0.556 | 0.1525 | -0.524 | 0.1827 |
| HER2 | Boltz-2 | 7 | 0.683 | 0.0907 | 0.429 | 0.3374 |
|  | RTMScore | 7 | -0.476 | 0.2806 | -0.571 | 0.1802 |
|  | MM/PBSA | 7 | 0.141 | 0.7634 | 0.143 | 0.7599 |
|  | MM/GBSA | 7 | -0.331 | 0.4678 | -0.036 | 0.9394 |
| KIT | Boltz-2 | 8 | 0.960 | 0.0002 | 0.905 | 0.0020 |
|  | RTMScore | 8 | -0.629 | 0.0949 | -0.810 | 0.0149 |
|  | MM/PBSA | 8 | 0.673 | 0.0674 | 0.524 | 0.1827 |
|  | MM/GBSA | 8 | 0.552 | 0.1564 | 0.500 | 0.2070 |
| PDGFRA | Boltz-2 | 8 | 0.942 | 0.0005 | 0.857 | 0.0065 |
|  | RTMScore | 8 | -0.627 | 0.0963 | -0.571 | 0.1390 |
|  | MM/PBSA | 8 | 0.677 | 0.0651 | 0.619 | 0.1017 |
|  | MM/GBSA | 8 | 0.596 | 0.1189 | 0.310 | 0.4556 |
| SRC | Boltz-2 | 8 | 0.973 | 4.60e-05 | 0.833 | 0.0102 |
|  | RTMScore | 8 | -0.656 | 0.0776 | -0.714 | 0.0465 |
|  | MM/PBSA | 8 | 0.392 | 0.3366 | 0.429 | 0.2894 |
|  | MM/GBSA | 8 | 0.249 | 0.5523 | 0.357 | 0.3851 |

**Table S18. Effects of sequence-context extension on predicted binding probability and kinase conformational states in EGFR inhibitor systems.** Predicted binding‑probability (BP) values are shown for different sequence‑input definitions, together with the change in BP relative to the kinase‑domain‑only (KD) model (ΔBP vs KD). αC‑helix and DFG conformational states were assigned using the distance‑based criteria described in the Methods section. IN/OUT classifications correspond to αC‑helix positioning and DFG‑motif orientation, respectively.

| Inhibitor | Region | BP | ΔBP vs KD | αC | DFG |
| --- | --- | --- | --- | --- | --- |
| Gefitinib | KD | 0.46 | 0.00 | IN | IN |
|  | (+)-KD | 0.50 | 0.04 | IN | IN |
|  | KD-(+) | 0.81 | 0.35 | IN | IN |
|  | (+)-KD-(+) | 0.82 | 0.36 | IN | IN |
| Lapatinib | KD | 0.30 | 0.00 | OUT | OUT |
|  | (+)-KD | 0.36 | 0.06 | OUT | OUT |
|  | KD-(+) | 0.69 | 0.39 | OUT | OUT |
|  | (+)-KD-(+) | 0.59 | 0.29 | OUT | OUT |
